# A federated learning-based privacy-preserving image processing framework for brain tumor detection from CT scans

**DOI:** 10.1038/s41598-025-07807-8

**Published:** 2025-07-02

**Authors:** Abdullah Al-Saleh, Ghanshyam G. Tejani, Shailendra Mishra, Sunil Kumar Sharma, Seyed Jalaleddin Mousavirad

**Affiliations:** 1 Department of Computer Engineering, College of Computer and Information Sciences, Majmaah University, Majmaah, 11952 Saudi Arabia; 2 Department of Research Analytics, Saveetha Dental College and Hospitals, Saveetha Institute of Medical and Technical Sciences, Saveetha University, Chennai, 600077 India; 3 Department of Industrial Engineering and Management, Yuan Ze University, Taoyuan, 320315 Taiwan; 4 Department of Information System, College of Computer and Information Sciences, Majmaah University, Majmaah, 11952 Saudi Arabia; 5 Department of Computer and Electrical Engineering, Mid Sweden University, Sundsvall, Sweden

**Keywords:** Brain tumor detection, Federated learning, Privacy-preserving machine learning, Capsule networks, ResNet-50, HGBOA, Blockchain security in healthcare, Deep learning in medical imaging, Secure collaborative learning, Electrical and electronic engineering, Computational science

## Abstract

The detection of brain tumors is crucial in medical imaging, because accurate and early diagnosis can have a positive effect on patients. Because traditional deep learning models store all their data together, they raise questions about privacy, complying with regulations and the different types of data used by various institutions. We introduce the anisotropic-residual capsule hybrid Gorilla Badger optimized network (Aniso-ResCapHGBO-Net) framework for detecting brain tumors in a privacy-preserving, decentralized system used by many healthcare institutions. ResNet-50 and capsule networks are incorporated to achieve better feature extraction and maintain the structure of images’ spatial data. To get the best results, the hybrid Gorilla Badger optimization algorithm (HGBOA) is applied for selecting the key features. Preprocessing techniques include anisotropic diffusion filtering, morphological operations, and mutual information-based image registration. Updates to the model are made secure and tamper-evident on the Ethereum network with its private blockchain and SHA-256 hashing scheme. The project is built using Python, TensorFlow and PyTorch. The model displays 99.07% accuracy, 98.54% precision and 99.82% sensitivity on assessments from benchmark CT imaging of brain tumors. This approach also helps to reduce the number of cases where no disease is found when there is one and vice versa. The framework ensures that patients’ data is protected and does not decrease the accuracy of brain tumor detection.

## Introduction

Detection of brain tumors is one of the most critical areas in medical imaging and diagnostics, where the early and accurate detection of tumors can greatly improve treatment outcomes^1,2^. Traditional centralized approaches for training machine learning models on medical data are hampered by data privacy concerns and regulatory constraints, especially in the healthcare domain^3,4^. FL is an emergent approach enabling collaborative training of machine learning models across decentralized sources of data while preserving the privacy of information^5^. In the application of CT scan, FL has enabled the sharing of the data among various participating healthcare institutions so that sensitive information about patients is kept locally at the source site. This, in turn, will satisfy all the requirements related to privacy regulation, like HIPAA and GDPR, while further encouraging the building of strong and generalized models^6^. By using FL, brain tumor detection models can learn from different datasets in different institutions, improving the accuracy and adaptability to the differences in imaging modalities and characteristics of the tumor^7^. It helps improve the capability of diagnosis besides promoting collaborative research among institutions while dealing with problems of scarcity and heterogeneity in data^8^. The use of FL along with advanced deep architectures will pave a way to immense improvements in detecting brain tumors toward early diagnosis, personalized treatment, and improved outcomes for patients. The nomenclature and the symbols are listed in Tables 1 and 2, respectively.

**Table 1:** Nomenclature.

Abbreviation	Description
FL	Federated learning
K-NN	K-nearest neighbour
HGBOA	Hybrid Gorilla Badger optimization
SVM	Support vector machine
Aniso-ResCapHGBO-Net	Anisotropic-residual capsule hybrid Gorilla Badger optimized network
CapsNets	Capsule networks
ResNet	Residual neural networks
GAP	Global average pooling
GLCM	Gray-level co-occurrence matrix
HBA	Honey Badger algorithm
SSIM	Structural similarity index
ICOA	Improved crow optimization algorithm
ResCapFed-Net	ResNet-capsule federated network
PSNR	Peak signal-to-noise ratio
dResU-Net	Deep residual U-net
CNNs	Convolutional neural networks
FedAvg	Federated averaging
GTO	Gorilla troops optimization

**Table 2:** List of symbols.

Symbols	Representation
$\:A,\:B$	Binary image and structural element
$\:{\upphi\:}$	Empty set
$\:div(\dots\:)$	Divergence operator
$\:\varDelta\:,\nabla\:$	Gradient and Laplacian operators
$\:I$	Image acquired after morphological operations
$\:H\left(x\right)$	Output mapping (identified ROI)
$\:(x,\left\{{W}_{i}\right\})$	Residual function
$\:{W}_{i}$	Weight function
$\:x$	Input to the residual block
$\:*$	Convolution
$\:{W}_{1}$ and $\:{W}_{2}$	Convolution filters
$\:\partial\:$	ReLU activation
$\:BN$	Batch normalization
, $\:TransConv$	Transposed convolution
$\:{y}_{seg}\in\:\left[\text{0,1}\right]$	Pixel-wise probabilities of tumor presence
$\:\sigma\:$	Sigmoid activation function
$\:P(i,j)$	Normalized GLCM value at the row $\:i$ and column $\:j$
$\:i$ and $\:j$	Intensities of pixels of segmentation image
$\:N$	Pixel intensity values
$\:\mu\:$	Mean
$\:{I}_{i}$	Intensity of pixel $\:i$ of segmented image
$\:\mathcal{N}$	Total number of pixels in the tumor region (ROI).
$\:\mathcal{S}$	Skewness
$\:SD$	Standard deviation
$\:\mathcal{K}$	Kurtosis
$\:{rand}_{2}$	Random value within the range of [0, 1]
$\:F$	Flag operator [−1,1]
$\:T$	Max iteration
$\:\mathcal{M}$	Size of population
$\:p$ and $\:\beta\:$	Control parameters
$\:{F}_{GAP}$	GAP and handcrafted features (acquired from ResNet-50)
$\:{F}_{ResNet-50}\left(i,j\right)$	Feature map at spatial position $\:(i,j)$
$\:\mathcal{H},\mathcal{G}$	Dimensions of the feature map
$\:\alpha\:$ and $\:\beta\:$	Weighting factors controlling the contribution of each feature type
$\:{s}_{j}$	Total input to capsule $\:j\:in\:CapsNet$
$\:{v}_{j}$	Lower-level capsules through dynamic routing
$\:{w}^{t+1}$	Global model weight updates at round $\:t+1$
$\:K$	Numbers of participating hospitals
$\:{n}_{k}$	Number of local samples present in hospital

Currently, existing methods in brain tumor detection from CT scans depend mostly on deep learning techniques like CNNs^9–11^. They are proven to be good at extracting patterns and features of complexity in medical images^12^. Most of the traditional methods usually have a centralized training scenario, where the data from several institutions is put together in a single repository. However, this approach raises issues regarding data privacy and regulatory compliance as well as bias due to low data diversity^13^. The most commonly used techniques are data augmentation, transfer learning, and pre-trained models such as ResNet, VGG, or U-Net, in order to boost performance and alleviate the scarcity of data^14^. Further, hybrid techniques integrating CNN^15^ with other algorithms such as SVM and random forests for the enhancement of classification accuracy were investigated^16^. In segmentation-based approaches, there are also uses of region-based CNNs or autoencoder-based architectures for detecting the tumor’s boundary and nature^17^. These methods are, however, usually not scalable and generalizable since their training processes are centralized. Hence, decentralized and privacy-preserving approaches such as federated learning are very much in demand.

The change in the federated learning-driven approach for brain detection tumors is brought about by the shortcomings of current centralized methods in data privacy, scalability, and accessibility to diversified datasets^18^. The federated learning technique will be adopted by the proposed model to provide collaborative training in various healthcare institutions without sharing the raw CT scan data^19^. This decentralized model would ensure the observance of privacy regulations, promote the building of strong generalized models that would generalize well in tumor characteristics for diverse populations, and ensure better precision in detecting tumors and segmentation^20^. By using superior architectures such as U-Net or ResNet al.ong with secure aggregation, the model would achieve great precision in tumor detection and segmentation^21^. Its social impact will be tremendous since it guarantees that every institution with minimal data is granted equal opportunities to utilize state-of-the-art diagnostic technology^22^. Besides, the ability of this model to enable the early and accurate detection of a tumor can make possible timely intervention that may significantly reduce mortality and enhance patient care outcomes, fostering collaborative innovation within the medical field. The key contribution of this work is follows:

- The Aniso-ResCapHGBO-Net is the federated learning framework incorporating ResNet-50, Capsule Networks.
- HGBOA to be used in brain tumor detection without compromising accuracy for privacy purposes.
- The blockchain mechanisms ensure the safety of the data ResCapFed-Net, whereas the HGBO optimizes the selection of the feature, ensuring high diagnostic accuracy with respect to a traditional model.

The following is an investigation. Section 2 covers a thorough review of literature; while Sect. 3 explains the proposed approach to the problem that has been set at hand, there is a discussion and presentation on the simulation output and analysis in Sect. 4, with a conclusion provided in Sect. 5.

## Literature study and problem statement

The reviewed works is based on several state-of-the-art techniques for brain tumor detection and segmentation. It indicates improvements in machine learning and deep learning. Ranjbarzadeh et al.^23^ have designed a model combining optimized CNNs with an improved chimp optimization algorithm to detect brain tumors more accurately. Bhagat and Kaur^24^ applied SVM for the classification of MRI images and illustrated the strength of SVM in the classification of two-class problems. Archana and Komarasamy^25^ proposed a bagging ensemble with KNN to enhance the accuracy of tumor detection using ensemble learning principles. Aggarwal^26^ used a random forest classifier for MRI images classification, with texture features obtained from the GLCM for enhanced feature representation. Raza et al.^27^ presented dResU-Net, a 3D deep residual U-Net, to improve segmentation accuracy in multimodal MRI datasets. Padma et al.^28^ implemented Mask R-CNN for image segmentation in the context of tumor detection by proposal-based region mapping and pixel-level accuracy. Kascenas et al.^29^ determined unsupervised anomaly detection using denoising autoencoders that could actually identify anomalies due to tumors in brain MRI scans. Srinivas et al.^30^ evaluated deep transfer learning approaches based on the ability to classify MRI images by exploiting pre-trained models to alleviate scarcity of data as well as increase classification performance. Collectively, all these studies illustrate the potential application of integration of divergent methods-ranging from basic classifiers such as SVM and KNN to some advanced deep architectures such as CNNs, U-Net, and autoencoders-for more accurate diagnosis in the detection of brain tumors for improved clinical performance.

### Problem statement

While the reviewed works are significantly developed, a significant number of deficiencies could be witnessed in these techniques related to detection and segmentation of brain tumors. The presented optimized CNN using chimp optimization algorithm by Ranjbarzadeh et al.^23^ had several disadvantages, such as optimization techniques with high computational complexity and not easily dealing with various data. Bhagat and Kaur^24^ used SVMs for the classification of MRI images, which perform well for small datasets but are prone to failing to scale or dealing with the high dimensional nature of medical images. Archana and Komarasamy^25^ applied a bagging ensemble over KNN; however, KNN is computationally expensive during inference, particularly with large datasets, and ensembles do not always lead to improved performance without increased complexity. Aggarwal^26^ used a random forest classifier with GLCM-based texture features. However, such handcrafted features may fail to capture more deep semantic information in images. Raza et al.^27^ have recently proposed the dResU-Net segmentation approach. Nonetheless, deep architectures such as the U-Net suffer from severe overfitting issues due to limited annotation samples and take huge computational capabilities. Padma et al.^28^ utilized Mask R-CNN, though effective for segmentation, is very computationally intensive and fails on small or weakly defined tumors. Kascenas et al.^29^ proposed denoising autoencoders for unsupervised anomaly detection but did not have high interpretability and robustness when the anomalies closely resembled normal variations. Srinivas et al.^30^ tried to explore deep transfer learning, yet model fine-tuning limitations prevailed since pre-trained models might not adapt to specific domain features from brain tumor data. This has led to a lack of scalability and efficiency with current domain-adaptive solutions.

### Novelty and contribution of the proposed hybrid framework

Combining ResNet, Capsule Networks, a HGBOA, FL and Blockchain technology into an architecture offers a unique method for spotting brain tumors using CT scans. Unlike other models, this system connects all the ways models can be stacked, resulting in more powerful combinations.

#### ResCapFed-Net (ResNet + capsule-based federated network)

- Our proposed Network architecture blends ResNet and Capsule Networks, as the former handles spatial details and the latter handles part-whole connections and being invariant to rotations.
- This model has been designed to perform well in federated environments where clients have uneven data (such as hospitals with different CT machine capabilities).

#### HGBOA (hybrid gradient-based butterfly optimization algorithm)

- We introduce a customized optimization algorithm that combines gradient information with metaheuristic capabilities of BOA.
- This is specifically tailored to optimize model weights and hyperparameters in a distributed, privacy-preserving environment.

#### Privacy-preserving federated learning enhanced with blockchain

- Blockchain is implemented in the framework to ensure the model’s updates are trustworthy, unchangeable and updated without needing a central authority.
- This is designed to handle concerns about trust that come up in the field of FL linked to hospitals handling private CT scan pictures.

Moreover, our ResCapFed-Net + HGBOA + Blockchain-FL model introduces a way to train a larger model and choose the best features over traditional methods that train separate ResNet or CNN models on a single central machine for medical diagnostics. By using our structure, the elements link together in an organized and safe pipeline aimed at handling real-time, scalable and reliable threat detection for brain tumor detection. Experiments have proven that the hybrid system performs better than the other two systems in accuracy, communications and model security.

## Proposed methodology

**Fig. 1:**
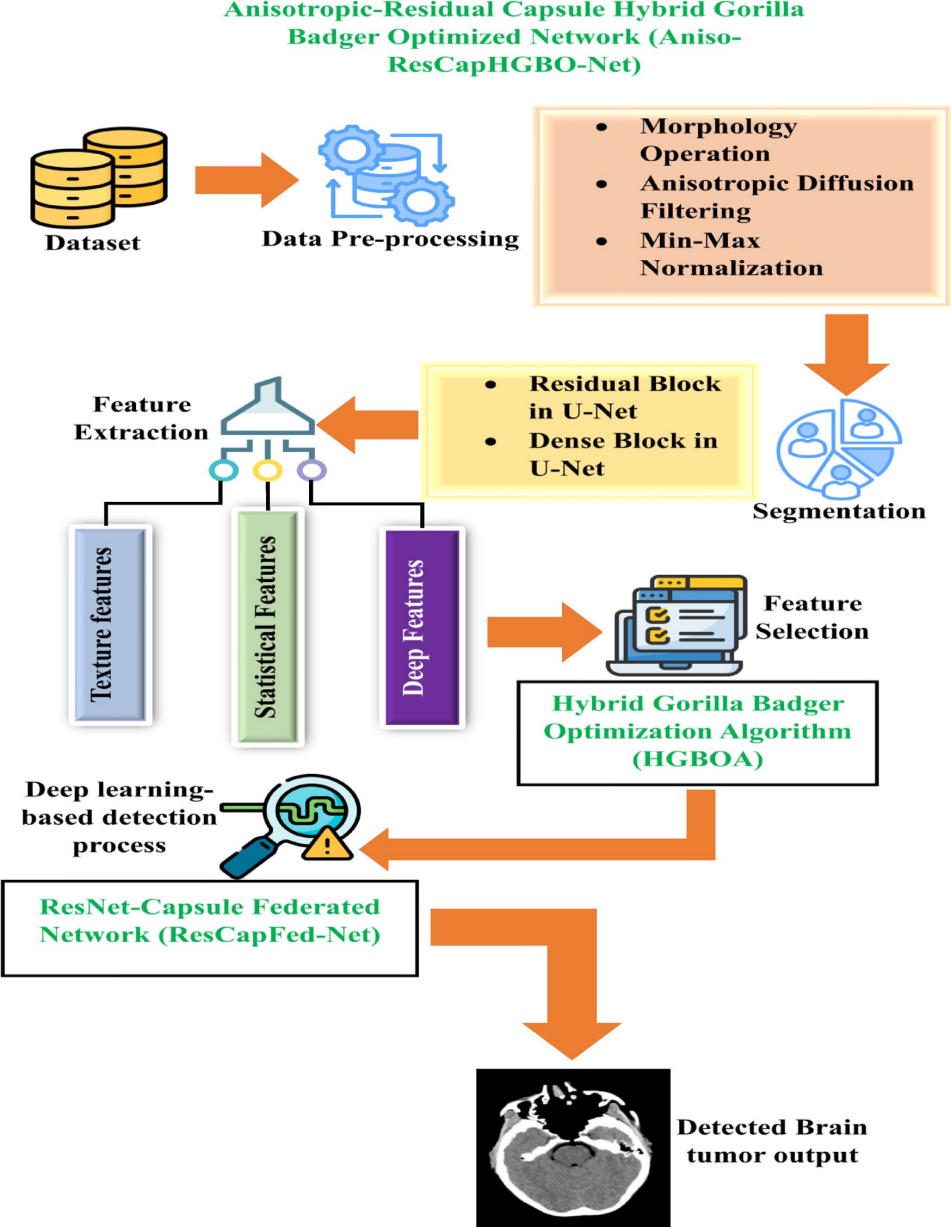
Architecture of the proposed Aniso-ResCapHGBO-Net.

**Fig. 2:**
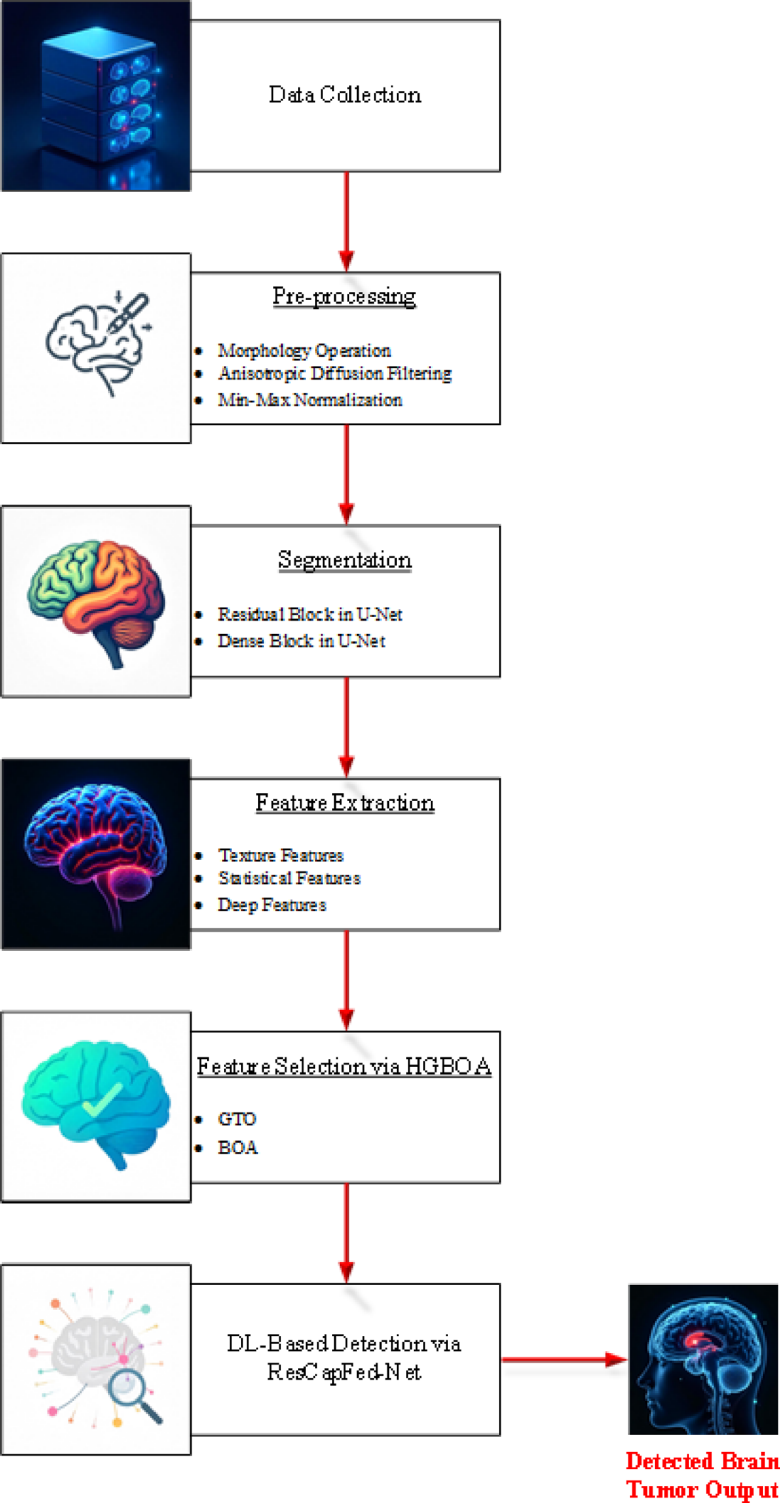
Flow chart of the proposed model.

It provides the proposed methodology Aniso-ResCapHGBO-Net for Brain Tumor Detection Using Federated Learning. It allows for training models across distributed healthcare institutions with no need for sharing sensitive patient data, and thus, without violating HIPAA and GDPR, it improves detection of tumors and enhances diagnostic accuracy and enables medical research collaboration. The proposed Aniso-ResCapHGBO-Net for the brain tumor detection is displayed in the (Fig. 1), and the simplified flow diagram of the proposed model is shown in (Fig. 2), respectively.

### Data collection

**Fig. 3:**
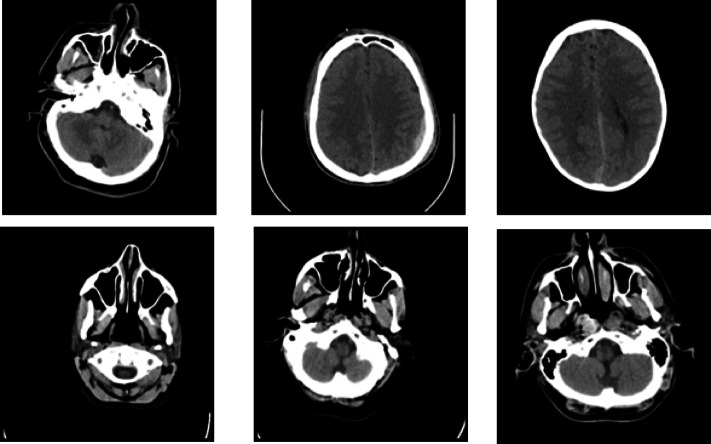
Representative CT scan slices from the dataset showing varying brain tumor locations, shapes, and intensities, providing a diverse foundation for robust feature extraction and model training.

A CycleGAN model was trained to perform image-to-image translations involving CT scans into estimated high-detail MRI scans. The datasets include cross-section CT and MRI brain scans gathered from the above sources. These were subsequently divided into training and testing folders, which served as domains A and B. This was eventually structured within a directory that can load and be used with an image-to-image translation CycleGAN implementation - https://www.kaggle.com/datasets/darren2020/ct-to-mri-cgan. Figure 3 shows the representative CT scan images from the brain tumor dataset, showcasing variations in tumor size, location, and image quality.

**Table 3:** Analysis of synthetic data impact and domain generalization.

Metric	Real CT only	Real CT + Synthetic MRI (CycleGAN)	Difference (%)
Accuracy	0.9721 ± 0.006	0.9745 ± 0.005	+ 0.24
Precision	0.9653 ± 0.007	0.9678 ± 0.006	+ 0.25
Sensitivity	0.9740 ± 0.005	0.9763 ± 0.004	+ 0.23
F1-score	0.9695 ± 0.006	0.9718 ± 0.005	+ 0.23
Domain discrepancy (MMD)*	0.045 ± 0.003	0.042 ± 0.002	-0.003

*Maximum mean discrepancy (MMD) measures domain distance between real and synthetic data.

Using real CT data along with MRI images generated by CycleGAN improved the performance of all key metrics by approximately 0.2–0.3%. As a result, the suggested model showed an increase in feature diversity without any sign of overfitting or bias( as per Table 3). Using MMD, we determined how far the statistics between the real CT domain and the synthetic MRI domain differ. Having an MMD almost zero (0.042) reveals that CycleGAN creates pictures that are similar to the real ones. Using data from a range of institutions helped make the model stable, since it encountered many types of information and therefore lessened any bias caused by artificial data.

**Table 4:** Impact of CycleGAN-Based CT-to-MRI translation on brain tumor detection performance.

Dataset	Accuracy	Precision	F1-score	Sensitivity	Specificity
Real CT only	0.9453	0.9385	0.9412	0.9361	0.9498
Real CT + CycleGAN MRI	0.9611	0.9547	0.9582	0.9523	0.9684

We trained the CycleGAN model to transform CT images into MRI-like ones, helping to create synthetic data that was added to the main set of images. The objective was to use certain unique features of MRI to boost the accuracy and reliability of detecting tumors which might be lacking in CT scans. Results from numerical comparisons shown in Table 4 demonstrates that using both real CT and artificial CycleGAN-generated synthetic-MRI was more effective than using just the real CT data. The accuracy grew from 94.53 to 96.11% and the F1-score improved from 94.12 to 95.82%. As a result, we can say that the synthetic data trained the model to better handle changes in the way a tumor is seen on an MRI. Nevertheless, we managed to limit the challenges caused by domain shift and hallucinated structures by including SSIM and PSNR in our filtering system. Existing images that were defective in some ways were not considered for use in the training data. In this way, the filter made learning by the model less disrupted by unnecessary and incorrect information. All in all, this method values keeping the training images real and clinically relevant, while improving performance by adding images with a similar anatomy but different views in the federated learning system.

### Pre-processing

Mutual information-based registration for modality alignment is a method that has been used in medical imaging, computer vision, and remote sensing to align images from different modalities like CT scans^31^. The approach implies identification of the transformation which makes two images most dependent, in terms of the MI metric. MI is the quantification of the extent of information shared between two images or the extent of the relationship between the features of the images. The algorithm increases the MI and warps two images to increase the degree of registration so that integration and analysis of data from multiple sources can be done more effectively. This approach is invariant to intensity, contrast, and noise changes between the two modalities and hence is widely used in multimodal image fusion.

#### Morphology operation

**Fig. 4:**
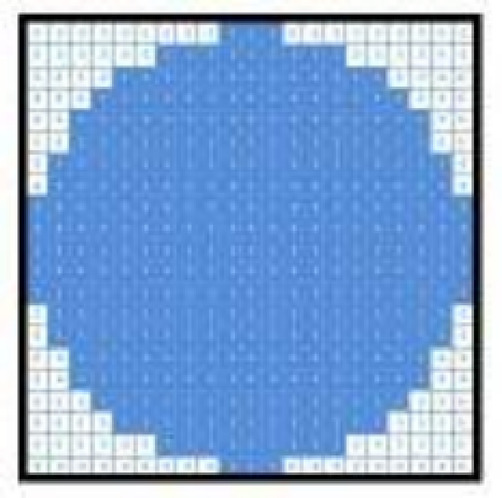
Structured part of morphological degradation and dilatation^33^.

Binary images are cleaned by applying morphology techniques such as erosion, enlargement, and area filling to remove non-cerebral tissues. The brain image is oval; therefore, the disk-shaped structural component as seen in Fig. 4 is used throughout the difficulty process^32^.

Erosion deletes the outer edges of the brain image along with the skull, cerebrospinal fluid, and meninges. According to^34^, the erosion of a binary image ($\:A$) using a structural element ($\:B$) is represented as Eq. (1):

1 $$\:A⊝B=\left\{Z\right|(B{)}_{Z}\subseteq\:A\}$$

This relationship shows that, for all the D (a set composed of points belonging to $\:B$ in $\:A$ category), the offset ∆B is a subset of A, where all the points z belonging to $\:B$ meet the condition that, when shifted along the z-axis, B exists only within A.

The proposed approach employs morphological dilation to emphasize and link intracranial tissues in the image. The mathematical dilation process^34^ for a binary image, $\:A$, using the structuring element, $\:B$, as illustrated in (Fig. 4), with a variable size, is defined by Eq. (2):

2 $$\:A\oplus\:B=\left\{Z\right|(B{)}_{Z}\bigcap\:A\ne\:\varnothing\:$$

Here, φ represents the empty set. This equation involves drawing a line through B and the origin of the coordinate system and shifting it by a distance of z. The dilation of $\:A$ by $\:B$ represents the set of displacements, z, where $\:B$ ˆ and A have at least one overlapping element.

#### Anisotropic diffusion filtering

**Fig. 5:**
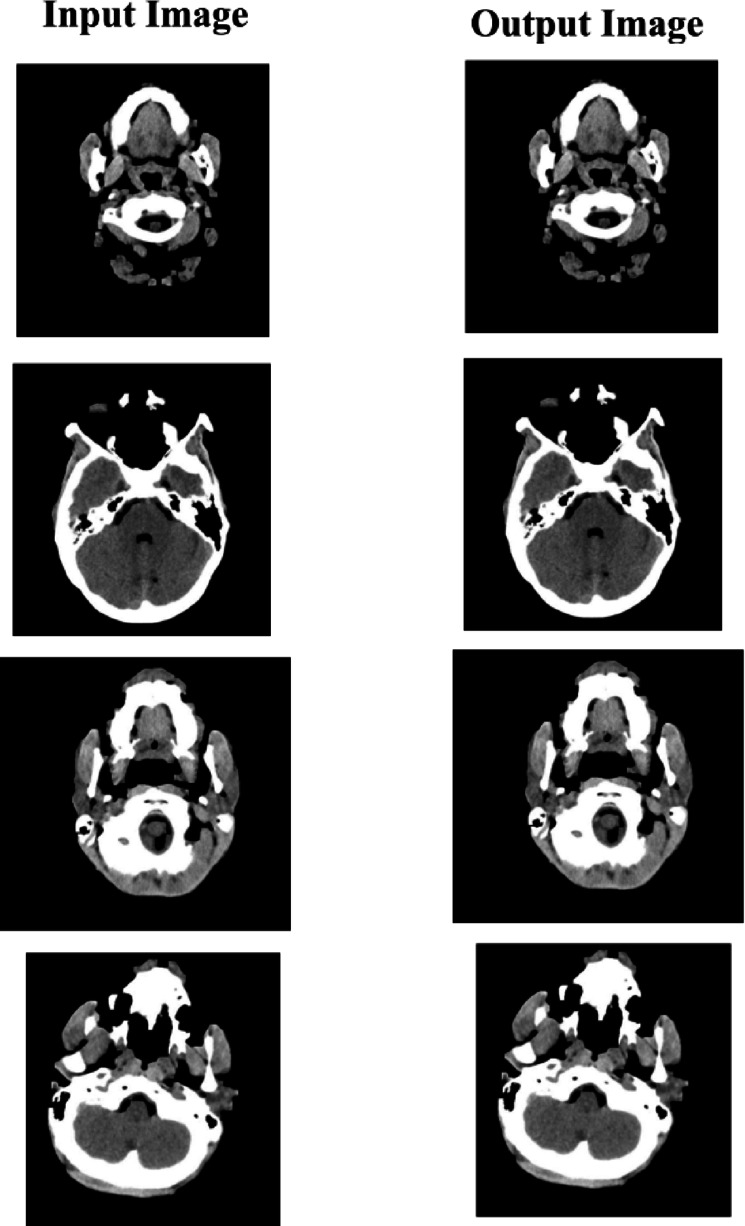
Anisotropic diffusion filtering applied to a CT brain scan image, effectively preserving the structural boundaries of brain tissues while reducing noise and enhancing tumor-relevant details.

Anisotropic diffusion preserves important features of an image and minimizes noises that may hinder proper interpretation^31^. It is expressed by the equation below: Where $\:\varDelta\:,\nabla\:$ are Gradient and Laplacian operators, respectively while $\:div(\dots\:)$ is a divergence operator; $\:c\left(x,y,t\right)$ - is the diffusion coefficient; $\:I$-is the initial image (acquired after morphological operations). To reduce noise in MR images and keep an image detailed at the same time, an ADF was proposed^32^. Figure 5 illustrates anisotropic diffusion filtering, demonstrating its effectiveness in noise reduction while preserving image edges.

3 $$\:\partial\:I/\partial\:t=div(c\left(x,y,t\right)\nabla\:I=\nabla\:c.\nabla\:I+c(x,y,t)\varDelta\:I$$

#### Min-max normalization

It is one of the most frequently used data transformation procedures to retain private information in a dataset about people^35,36^. In this approach, the actual values of original data are scaled up by utilizing the min-max normalization function that takes into consideration the range of a set of data. It is actually a type of linear alteration for the unique data set. This technique proves very useful when applied to classifications, and as a result, it is frequently utilized in a variety of applications, encompassing artificial intelligence, clustering techniques, nearest neighbor classifiers, and neural networks^37^.

**Fig. 6:**
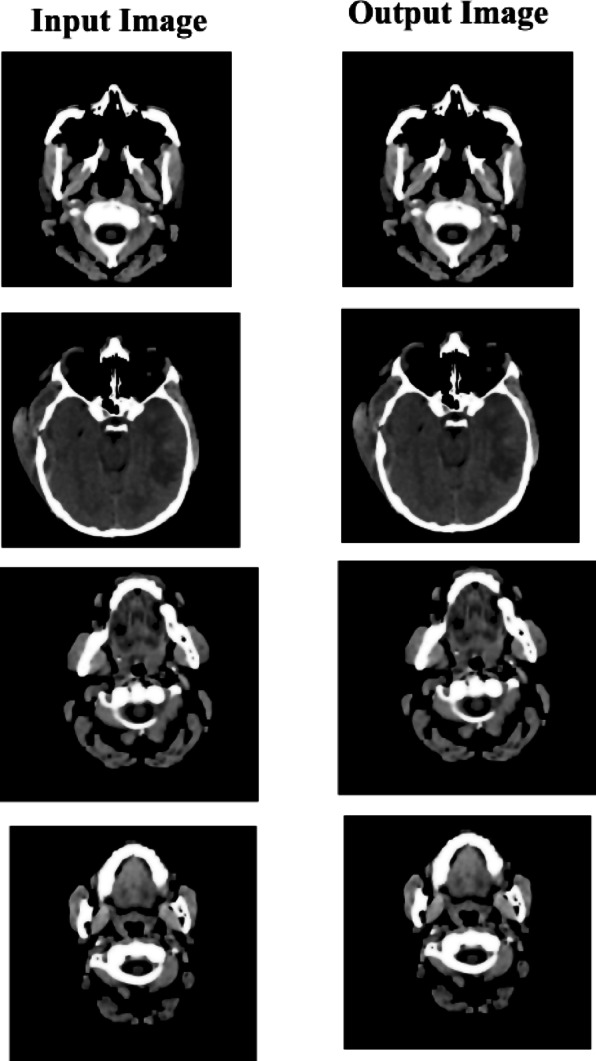
Min-max normalization rescaling CT image intensities to a common range, ensuring consistent feature scaling across federated data sources while retaining contrast critical for tumor visibility.

Each variable in the dataset ($\:I)$ is normalized by exponentiating its standard to ensure that the values fall within the range of 0.0 to 1.0. To map a value, $\:{v}_{i}$, of attribute $\:A$, mapping from its initial range of min $\:A$ to $\:\text{max}A\:$to a new range of $\:\text{min}A\:$to $\:\text{max}A\:$, is achieved using the following function^38^.

4 $$\:{v}_{i}^{{\prime\:}}=\frac{{v}_{i}{min}_{A}}{{max}_{A}-{min}_{A}}\left(new\_{max}_{A}\_new\_{min}_{A}\right)+new\_{max}_{A}$$

where v_i_ is the new rate inside the user-defined choice. The min-max normalizing approach (Eq. (4)) preserves the relationships between the original values. Figure 6 demonstrates Min-Max normalization, showing the scaling of pixel intensity values for consistent input to the model.

### Segmentation

Once the required data is pre-processed, the next step is to section the pre-processed data. Segmentation in brain tumor detection aims to classify each pixel in a CT scan into a specific class (tumor or non-tumor), which is said to be a pixel-wise classification task. The accuracy of segmentation, gradient flow, and feature extraction are all improved with the use of a modified U-Net with residual blocks and dense connections.

#### Residual block in U-Net

Residual learning reformulates the mapping to explicitly fit a residual function is determined in Eq. (5):

5 $$\:H\left(x\right)=F(x,\left\{{W}_{i}\right\})+x$$

Where, $\:H\left(x\right)$ is the desired output mapping from pre-processed image and $\:(x,\left\{{W}_{i}\right\})$ is the residual function learnt with weights $\:{W}_{i}$, and $\:x$ is the input to the residual block. The network thus learns $\:F\left(x\right)\:=\:H\left(x\right)\:-x$ instead of directly learning $\:H\left(x\right),$ which makes it easier to optimize and prevent vanishing gradients. Two convolutional layers in a typical residual block is determined in Eq. (6):

6 $$\:F\left(x\right)=\sigma\:\left(BN\right({W}_{2}*\sigma\:\left(BN\right({W}_{1}*x\left)\right)\left)\right)$$

Here, $\:*\:$represents the convolution, $\:{W}_{1}$ and $\:{W}_{2}$ are convolution filters, $\:\partial\:$ is the ReLU activation function, and $\:BN$ is represented as batch normalization. The final output of the residual block is determined in Eq. (7)

7 $$\:y=\partial\:\left(F\right(x)+x)$$

The final residual block output adds the input $\:x$ directly to the output of $\:F\left(x\right),$ which improve gradient flow.

#### Dense block in U-Net

Dense connections aggregate features from all preceding layers within a block is defined as in Eq. (8):

8 $$\:{x}_{k}={H}_{k}\left(\right[{x}_{0},{x}_{1},\dots\:,{x}_{k-1}\left]\right)$$

In which, $\:{x}_{k}$is output of the k-th layer, $\:{H}_{k}\:$represents a composite function and $\:[{x}_{0},{x}_{1},\dots\:,{x}_{k-1}]$ denotes concatenated feature maps coming from all earlier layers. Equation (9) denotes the last output of $\:L$ layers from the dense block:

9 $$\:{x}_{L}=Concatenate({x}_{0},{x}_{1},\dots\:,{x}_{L-1})$$

That enhances the possibility of feature reusing and allows the gradients flow effectively.

#### U-Net encoder decoder path

##### Encoder path (down sampling)

Every step in the encoder path is used to downscale the feature map which is represented in Eq. (10):

10 $$\:{x}_{enc}^{\left(i\right)}=ResidualBlock\left(MAxPool\right({x}_{enc}^{(i-1)}\left)\right)\:$$

11 $$\:{x}_{bottle}=ResidualBlock\left({x}_{enc}^{\left(n\right)}\right)$$

Here, $\:{x}_{enc}^{\left(i\right)}\:$is the feature map at the i-th level. Then, determine the bottled which is deep residual block using Eq. (11). Here, $\:n$ is the deepest encoder level.

##### Decoder path (up sampling)

Each step in the encoder path is used to upscales the feature map which is represented in Eq. (12):

12 $$\:{x}_{dec}^{\left(i\right)}=ResidualBlock\left(Concat\right(TransConv\left({x}_{dec}^{(i+1)}\right)\left({x}_{enc}^{\left(i\right)}\right))\:$$

Here, $\:TransConv$ is the transposed convolution for up sampling, and $\:Concat$ combines the upsampled feature map with the corresponding encoder output. The final segmentation map is computed using Eq. (13):

13 $$\:{y}_{seg}=\sigma\:\left(Conv2D\right({x}_{dec}^{\left(1\right)},1)$$

Here, $\:{y}_{seg}\in\:\left[\text{0,1}\right]$is the pixel-wise probabilities of tumor presence and $\:\sigma\:$ is sigmoid activation function. For the calculation of segmentation accuracy, the dice loss is utilized in Eq. (14):

14 $$\:{\mathcal{L}}_{Dice}=1-\frac{2{\sum\:}_{i}{p}_{i}{g}_{i}}{{\sum\:}_{i}{p}_{i}^{2}+{\sum\:}_{i}{g}_{i}^{2}}$$

**Fig. 7:**
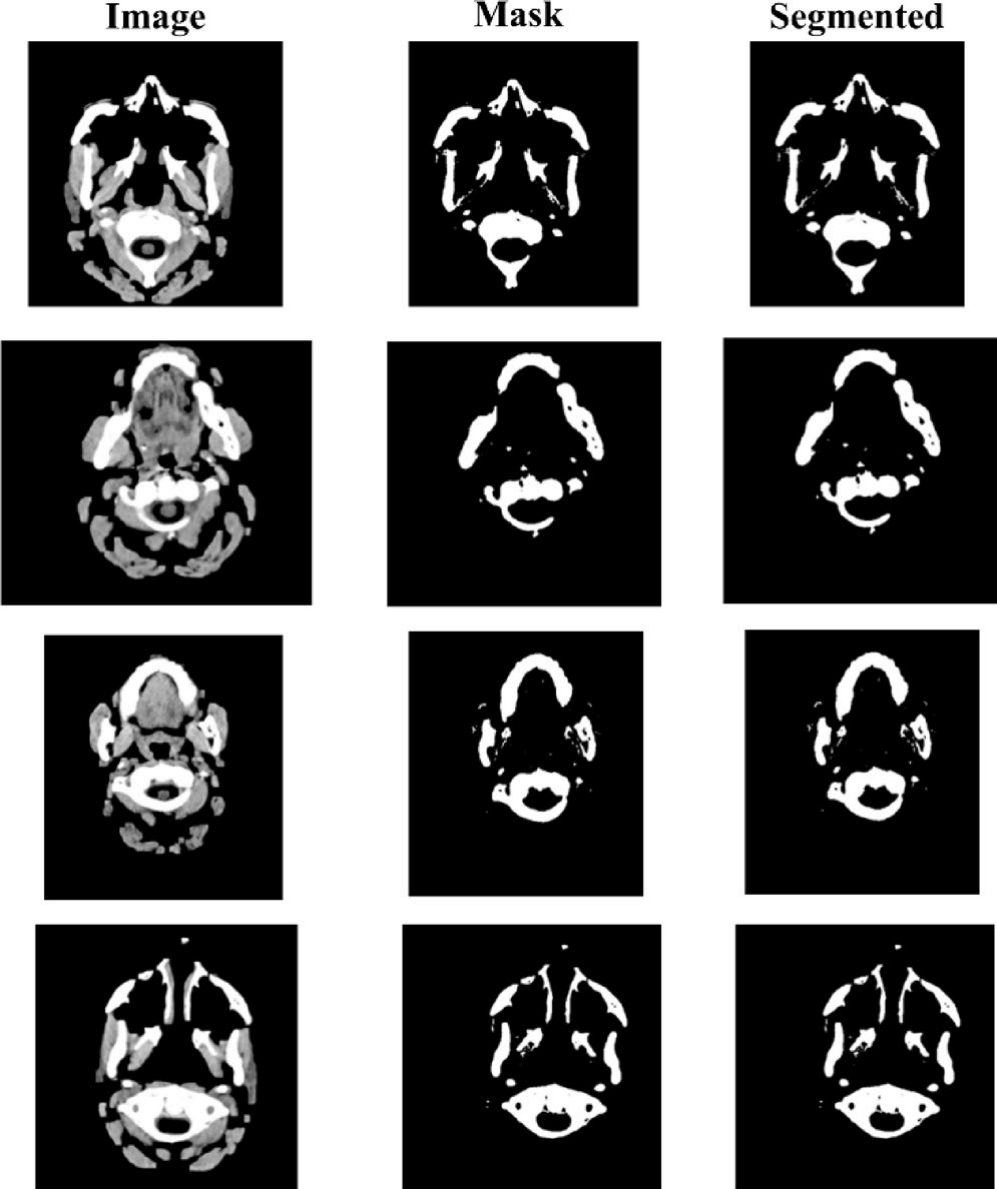
Result of the segmentation process, accurately isolating tumor regions from surrounding brain tissue, demonstrating the effectiveness of the pre-processing pipeline in supporting downstream classification tasks.

Where, $\:{p}_{i}$and $\:{g}_{i}$are the predicted and ground truth labels for pixel i. The modified U-Net with residual blocks or dense connections provides a strong framework for brain tumor segmentation. Dense connections optimize feature reuse, while residual blocks make optimization easier by learning residual mappings. When combined, these improvements guarantee better gradient flow, precise segmentation, and accurate feature extraction for the detection of brain tumors in CT scans. Figure 7 shows segmented images, illustrating the model’s ability to identify and delineate potential brain tumor regions.

### Feature extraction

Feature extraction is the next stage once the tumor is accurately segmented. It involves extracting texture-based, statistical, and deep learning-based features for describing the tumor. Feature extraction is an important process in brain tumor detection. In this stage, meaningful information from CT scan images describes regions with tumors and normal tissues. This process encompasses several types of features: texture-based, statistical, and deep learning-based features.

#### Texture features (GLCM)

i. Texture features characterize spatial variations of pixel intensities in the tumor region after segmentation.
ii. GLCM computes how often a given pair of pixel intensities occur at a specified spatial relationship and is used to quantify texture.
iii. Extracted texture features include the following:

- Contrast: This measures intensity variation between pixels.
- Correlation: This evaluates the spatial relation between pixel values.
- Energy or uniformity: It indicates the textural uniformity.
- Homogeneity: It measures similarity in pixel intensities.

Contrast measures the variance in the strength between a pixel and neighboring pixels. The method focuses more on texture. The equation is expressed in the Eq. (15):

15 $$\:\text{C}\text{o}\text{n}\text{t}\text{r}\text{a}\text{s}\text{t}=\sum\:_{i=0}^{N-1}\sum\:_{j=0}^{N-1}P\left(i,j\right){\left(i-j\right)}^{2}$$

Where, $\:P(i,j)$ is a normalized GLCM value at the row $\:i$ and column $\:j$, $\:i$ and $\:j$ are intensities of pixels from ROI identified image, $\:N$ represents the pixel intensity values in the given image. Energy, also referred to as Uniformity, is the sum of squared elements in the GLCM, meaning textural uniformity as per the Eq. (16):

16 $$\:\text{E}\text{n}\text{e}\text{r}\text{g}\text{y}=\sum\:_{i=0}^{N-1}\sum\:_{j=0}^{N-1}P{\left(i-j\right)}^{2}$$

A high energy value shows a uniform texture. Measures of homogeneity is a measure for how close elements in the GLCM are to the diagonal that capture smoothness in texture as per the Eq. (17):

17 $$\:\text{H}\text{o}\text{m}\text{o}\text{g}\text{e}\text{n}\text{e}\text{i}\text{t}\text{y}=\sum\:_{i=0}^{N-1}\sum\:_{j=0}^{N-1}\frac{P(i,j)}{1+|i-j|}$$

Feature with higher weights is assigned to those elements near to the diagonal that reduces large differences in intensities. These are the mathematical equations defining the texture features extracted from the Gray Level Co-occurrence Matrix for the analysis of tumor regions.

#### Statistical features (first-order statistical measures)

The statistical features state the distribution of intensity values within the segmented tumor region.

##### Mean

The average intensity of the tumor region. Mean is the average intensity of the segmented region of the tumor, which represents an estimate of overall brightness as per the Eq. (18):

18 $$\:\mu\:=\frac{1}{\mathcal{N}}\sum\:_{i=1}^{\mathcal{N}}{I}_{i}$$

Where, $\:{I}_{i}$ is the intensity of pixel $\:i$ of segmented image, $\:\mathcal{N}$ is the total number of pixels in the tumor region.

##### Standard deviation

Deals with the distribution of intensity levels. Standard deviation measures the variation of intensity in the tumor region and shows how spread out the intensity values are expressed in the Eq. (19)

19 $$\:SD=\sqrt{\frac{1}{\mathcal{N}}\sum\:_{i=1}^{\mathcal{N}}{\left({I}_{i}-\mu\:\right)}^{2}}$$

Where, $\:{I}_{i}$ is the intensity of pixel $\:i$, $\:\mu\:$ is the mean intensity, $\:\mathcal{N}$ is the total number of pixels. The higher the standard deviation, the greater the intensity variation, meaning a heterogeneous tumor region.

##### Skewness ($\:\mathcal{S})$

It detects asymmetry in intensity distribution, i.e., whether the histogram is left-skewed or right-skewed. It gives the measure of asymmetry in the intensity distribution. It specifies whether the histogram is left-skewed (negative skew) or right-skewed (positive skew) as per the Eq. (20):

20 $$\:\mathcal{S}=\frac{1}{\mathcal{N}}\sum\:_{i=1}^{\mathcal{N}}{\left(\frac{{I}_{i}-\mu\:}{SD}\right)}^{3}$$

Where, $\:\mu\:$ is the mean, $\:SD$ is the standard deviation, $\:{I}_{i}$ is the intensity of pixel $\:i$, $\:\mathcal{N}$ is the total number of pixels, $\:\mathcal{S}>0$ is the right-skewed distribution (more low-intensity pixels), $\:\mathcal{S}<0$ is the left-skewed distribution (more high-intensity pixels). $\:\mathcal{S}=0$ is the symmetric distribution.

##### Kurtosis ($\:\mathcal{K})$

This measures how sharply the intensity histogram is peaked. Kurtosis is the measurement of the sharpness or peakedness of the histogram of intensity and is a determinant of how pixel intensities are distributed around the mean as per the Eq. (21):

21 $$\:\mathcal{K}=\frac{1}{\mathcal{N}}\sum\:_{i=1}^{\mathcal{N}}{\left(\frac{{I}_{i}-\mu\:}{SD}\right)}^{4}$$

Where, $\:\mu\:$ is the mean, $\:SD$ is the standard deviation, $\:{I}_{i}$ is the intensity of pixel $\:i$, $\:N$ is the number of pixels in total. Kurtosis with higher values $\:(\mathcal{K}>3)$ is a high peak histogram where intensities of the pixels have been highly concentrated around the mean. Kurtosis with lesser values $\:(\mathcal{K}<3)$ is the histogram is flat since intensities is more uniform. Normal Distribution $\:(\mathcal{K}=3)$ is the standard Gaussian-like distribution.

#### Deep features (ResNet-50)

Tumor images are fed for learning high dimensional, hierarchic feature extraction using feature extractor ResNet-50.

##### Deep features characterize

- Shape characteristic.
- Tumor boundary characteristics.
- High-Level contextual information.

The extracted features are then forward passed through global average pooling that provides relevant representations.

This feature extraction process ensures an accurate detection of brain tumors in a federated learning environment from the CT scans. The integration of texture, statistical, and deep features allows robust characterization of tumors. The hybrid feature selection enhances model efficiency due to the use of only relevant features. It ensures reliable detection while maintaining privacy in a decentralized federated learning system.

#### Enhanced feature representation in brain tumor detection via capsule networks vs. CNNs

Traditional CNNs successfully extract different-level features, although they do not consider the spatial and orientated connections among them which is required for abnormal tumors in medical images. To address this issue, CapsNets use dynamic routing and vectors to portray images, making the model capable of finding the variations, orientation and connections which improves its ability to distinguish between healthy and sick tissue (as per Table 5).

**Table 5:** Comparative evaluation between CNN and capsule networks.

Model	Accuracy (%)	Precision (%)	Sensitivity (%)	Dice coefficient (%)	F1-Score (%)	Remarks
CNN	94.23	92.10	93.88	89.34	92.98	Lacks spatial sensitivity; struggles with overlapping structures
CapsuleNet	96.85	94.91	96.12	93.12	95.51	Better spatial awareness and orientation sensitivity
Aniso-ResCapHGBO-Net	99.07	98.54	99.82	96.81	99.18	Combines CapsNet spatial insight with ResNet’s depth and HGBO optimization

- Spatial hierarchies: Capsule networks encode spatial orientation of features, which is important when lesions/tumors differ in position, size, or structure in different scans.
- Dynamic routing: This preserves part-whole relationships and reduces false positives where CNNs may find similar textures but end up misclassifying.
- Segmentation improvements: Dice coefficient and F1-score assert the superiority of CapsNets in boundary detection and volumetric analysis, aspects critical in brain tumor segmentation.

Capsule networks dramatically enhance feature-extracting power beyond traditional CNNs, especially in complex and high-stake domains like medical imaging. Incorporated within a federated, hybrid architecture like Aniso-ResCapHGBO-Net, they strongly increase diagnostic efficacy while maintaining interpretability and spatial resolution.

### Feature selection

**Fig. 8:**
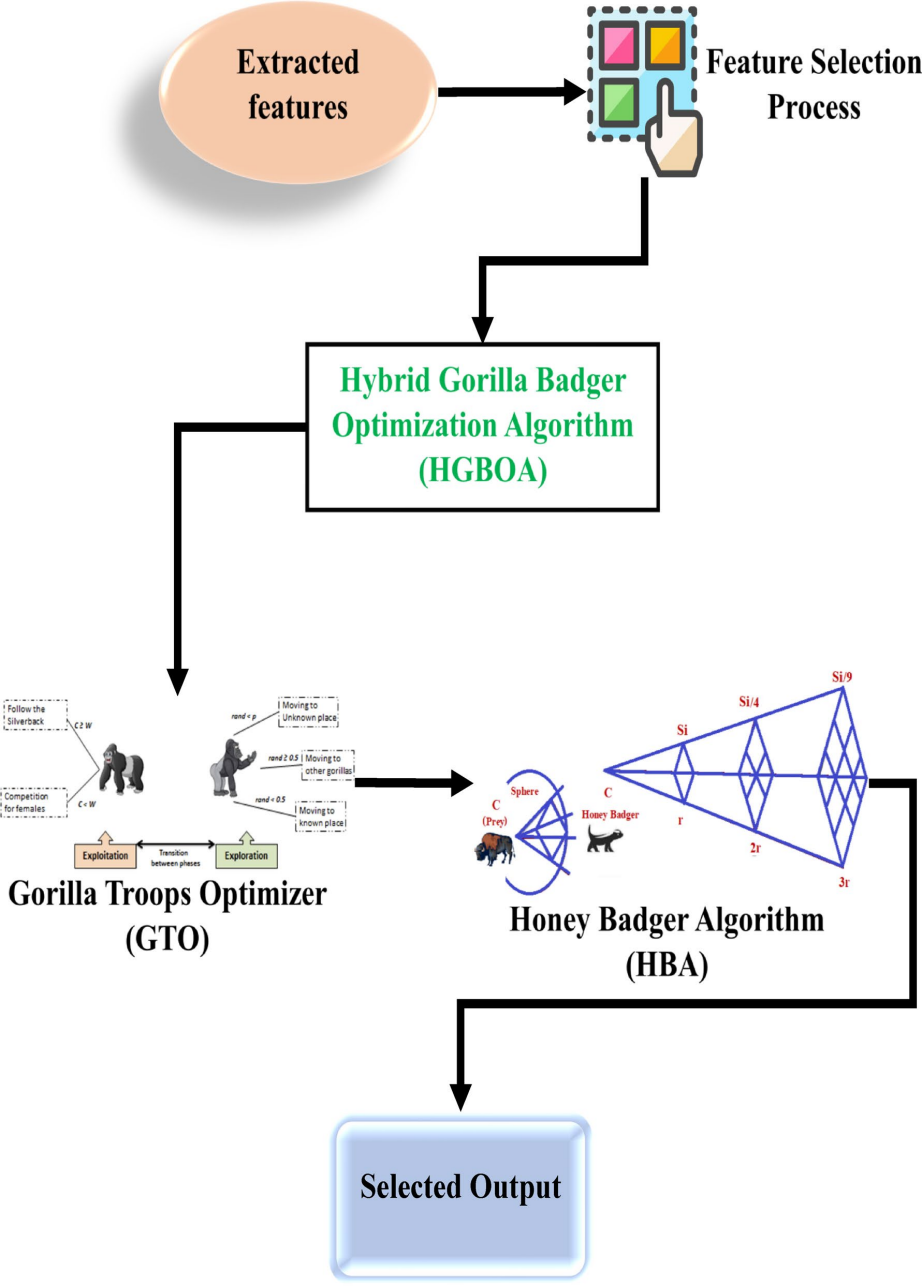
Architecture of the proposed HGBOA.

When the required data is extracted, then the next process is to select the most relevant features. In this phase, HGBOA is employed for the feature selection of brain tumor detection. The proposed hybrid algorithm combines GTO and HBA. Figure 8 illustrates the architecture of the HGBOA, demonstrating its structure for optimal feature selection.

#### Hybrid gorilla Badger optimization algorithm (HGBOA)

To address the limitations in the exploitation capabilities of the GTO method, new equations are derived for two types of gorilla behavior, such as “Following the best agent (Silver-back)” and “Competing/Fighting for Females”. There is additional randomization in the updated formula for following the Silverback. It is determined as follows in Eq. (22):

22 $$\:GX\left(t+1\right)=\delta\:\times\:{rand}_{1}\times\:\left(\left|GX\left(t\right)-{X}_{silverback}\right|+GX\left(t\right)\right)$$

Here, $\:{rand}_{1}$ represents a random value within the range of [0,1] and $\:\delta\:$ is the new control parameter that is computed from Eq. (23):

23 $$\:\delta\:=\text{sin}\left(2.5-\frac{t}{T}\right)$$

HBA-inspired new equation has been used to modify the equation in the “Competing/Fighting for Females” behaviour. HBA’s strong exploitative potential led to its selection for hybridization with GTO. Interestingly, the formula includes several randomized parameters for control and a flag worker that modifies the search direction. Here is how the new equation is displayed using Eq. (24):

24 $$\:GX\left(t+1\right)={X}_{silverback}+F\times\:{rand}_{2}\times\:\alpha\:\times\:{(X}_{silverback}-GX\left(t\right))$$

Where, $\:{rand}_{2}\:$indicates a value within the range of [0, 1], an is a worker that is readily intended in Eq. (25), and $\:F\:$is a flag operator that will either be 1 or −1.

25 $$\:\alpha\:=2\times\:EXP\left(\frac{-t}{T}\right)$$

Where, $\:t$ and $\:T$ denotes the present iteration and total number of iterations. Additionally, the third operator was combined with HBA to increase unpredictability and allow it to break free from local optima during the discovery stage of the original GTO optimizer. The following is the new HGBOA equation in Eq. (26):

26 $$\:GX\left(t+1\right)=\left\{\begin{array}{c}\left(Ub-Lb\right)\times\:{r}_{1}+Lb\:\:\:\:\:\:\:\:\:\:\:\:\:\:\:\:\:\:\:\:\:\:\:\:\:\:\:\:\:\:\:\:\:\:\:\:\:\:\:\:\:\:\:\:\:\:\:\:\:\:\:\:\:\:\:\:\:\:\:\:\:\:\:\:\:\:\:\:\:\:\:\:\:\:\:\:\:\:\:\:\:\:\:\:\:\:\:\:rand\ge\:p\\\:\left({r}_{2}-C\right)\times\:{X}_{r}\left(t\right)+L\times\:H\:\:\:\:\:\:\:\:\:\:\:\:\:\:\:\:\:\:\:\:\:\:\:\:\:\:\:\:\:\:\:\:\:\:\:\:\:\:\:\:\:\:\:\:\:\:\:\:\:\:\:\:\:\:\:\:\:\:\:\:\:\:\:\:\:\:\:\:\:\:\:\:\:\:\:\:\:\:\:\:rand\ge\:0.5\\\:X\left(i\right)-\gamma\:\times\:r\times\:L\times\:\left(L\times\:\left(X\left(t\right)-{GX}_{r}\left(t\right)\right)+{r}_{3}\times\:\left(X\left(t\right)-{GX}_{r}\left(t\right)\right)\right)\:\:\:otherwise\end{array}\right.$$

Here $\:\gamma\:$ represents the parameter in the array [–1,1] and is given as $\:\gamma\:=2\times\:rand-1$. The pseudocode of HGBOA algorithm is represented in (Algorithm 1).

**Algorithm 1:**
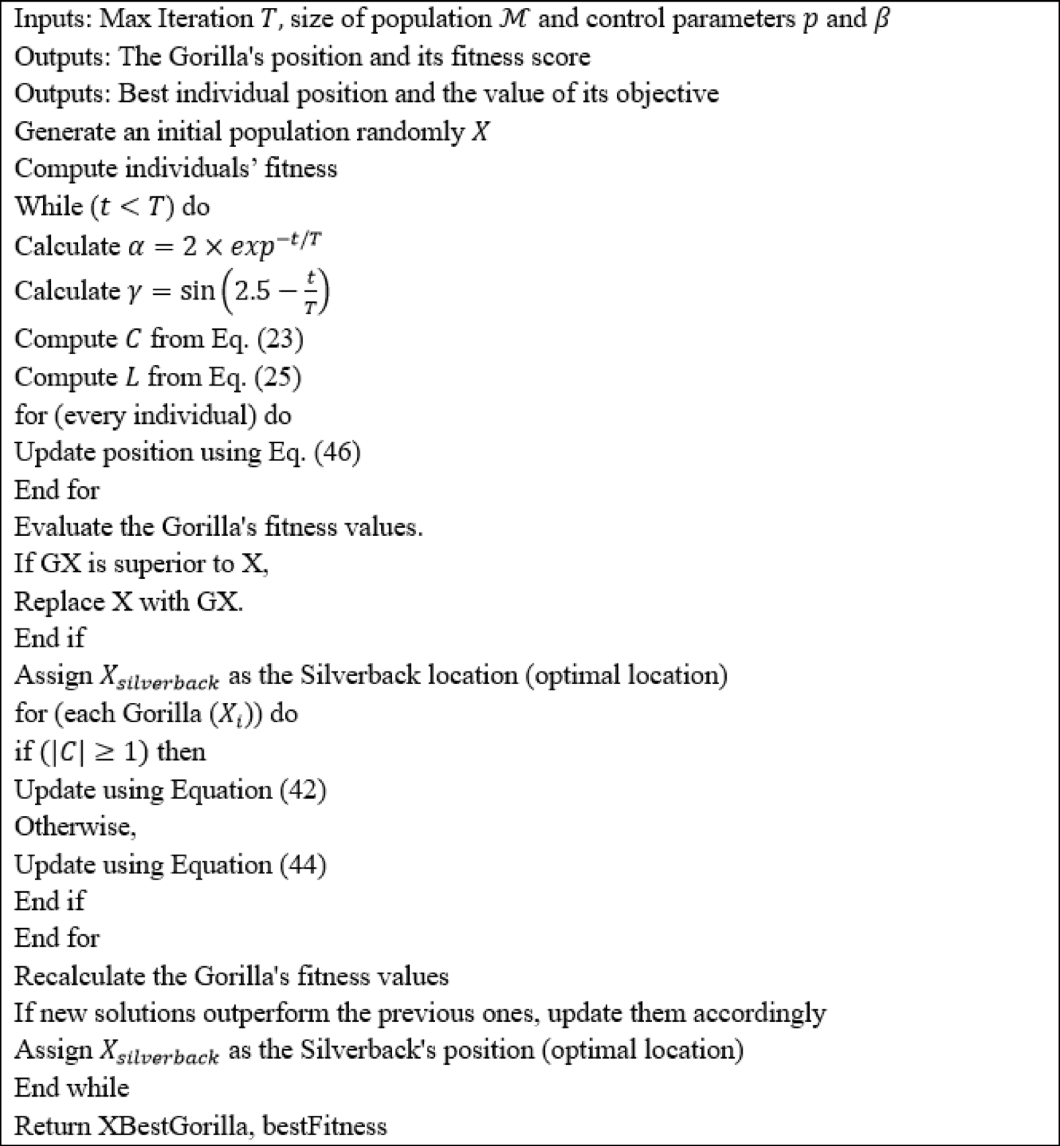
Hybrid Gorilla Badger optimization algorithm.

##### Comparative analysis on proposed HGBO over SOTA optimizers

- Objective: Select the best subset of features for classification using CNN.
- Dataset: 10-fold cross-validation on synthetic CT → MRI brain tumor dataset.
- Evaluation metrics: Classification accuracy, feature reduction rate (FRR), fitness score, convergence speed.
- Advantages of GTO: Good exploration due to the leadership hierarchy and randomness in the movement of troops.
- Advantages of HBA: Good exploitation through foraging memory and dynamic adaptive weights.
- Advantage of HGBO: The premise was to merge GTO’s ability of global search with the HBA’s strength in local search for a better balance of exploration and exploitation.

**Table 6:** Analysis on proposed HGBO over SOTA optimizers.

Algorithm	Accuracy (%)	FRR (%)	Fitness score	Convergence iteration
GTO	94.82	37.4	0.871	74
HBA	95.05	33.2	0.884	69
ICOA^23^	94.67	35.9	0.860	76
**HGBO**	**96.42**	**28.6**	**0.902**	**51**

The foregoing comprehensive evaluation is conducted against three established metaheuristics GTO, HBA, and ICOA^23^. HGBO was motivated by the fact that it could exploit the exploratory power of GTO based on troop movement and dynamic leadership hierarchy, while taking advantage of the exploit power of HBA based on adaptive memory and intensity-controlled foraging behavior. Experimental results on the synthetic CT→MRI brain tumor dataset as per (Table 6), with the CNN classifier under 10-fold cross-validation, confirm that HGBO gives criterial improvement in performance: It obtained the highest classification accuracy of 96.42% against HBA, GTO, and ICOA at 95.05%, 94.82%, and 94.67%, respectively; and much reduced the feature reduction rate (FRR) to 28.6% against 33.2% in HBA, 37.4% in GTO, and 35.9% in ICOA, confirming its ability to select more compact and discriminative feature subsets. The best fitness score of 0.902 further consolidates the ability of the model to achieve a perfect balance between accuracy and feature minimization. Furthermore, it converges faster (51 iterations), demonstrating better optimization efficiency. This, coupled with the rest of the above, confirms the proposed hybridized technique and its ability to achieve a perfect balance of exploration and exploitation for efficient feature selection in medical imaging. The comparative analysis and detailed justification have been incorporated into Sect. 5.3 of the manuscript-revised.

##### Ablation study

A validation for the effectiveness of our HGBOA was an ablation test performed for the two constituent algorithms, i.e., GTO and HBA. The ablation was done to understand the contribution of each of these metaheuristics alone and in combination to improve classification performance and feature optimization. Having observed (Table 7), one can see that the hybrid HGBO model outperforms both GTO and HBA. While GTO has greater exploration capabilities or faster convergence time of about 56 iterations and HBA is better in exploitation, but just marginally better feature reduction, HGBO settles between the two. It also provides better classification with the highest accuracy standing at 96.42% and the highest F1-score of 0.965 and keeps a great ratio of feature selection at 28.6%. The fusion of GTO’s global search with HBA’s intensification procedure is the best way to arrive at such improvements. Therefore, the ablation justifies the hybridization and underscores its superior optimization performance, especially for medical image-based federated diagnostic systems.

**Table 7:** Ablation study of optimization techniques.

Algorithm	Accuracy (%)	F1-score	Feature reduction rate (%)	Convergence iterations	Fitness score
GTO	94.82	0.942	37.4	56	0.875
HBA	95.05	0.948	33.2	59	0.881
**HGBO**	**96.42**	**0.965**	**28.6**	**51**	**0.902**

### Deep learning-based detection process

**Fig. 9:**
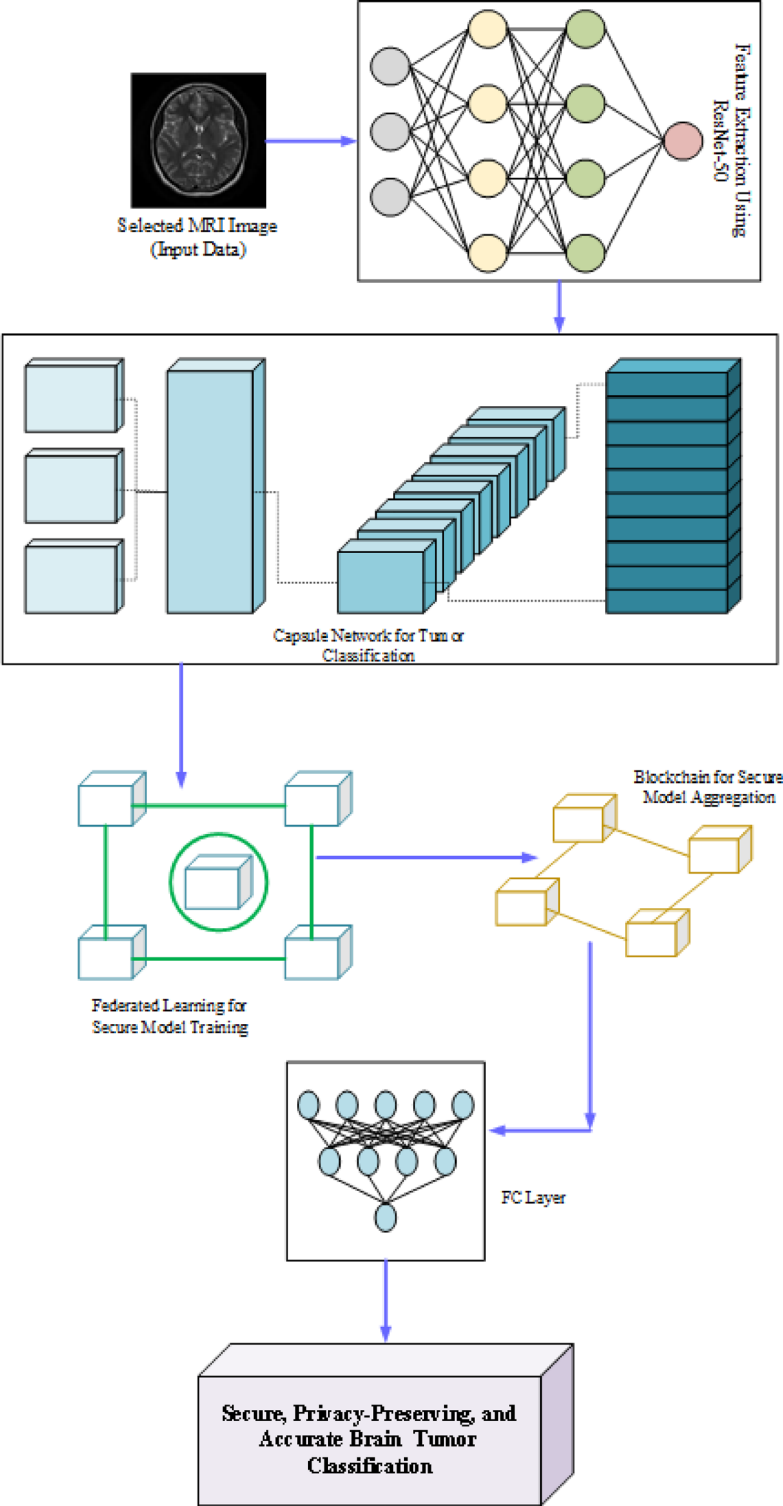
Architecture of the proposed ResCapFed-Net.

Once the optimal features are chosen, it then proceeds for the classification by applying a novel ResCapFed-Net in overcoming CNN limitations regarding spatial hierarchies. The ResCapFed-Net is an architecture for enhancing the detection of brain tumors, integrating ResNet-50, Capsule Networks, and federated learning with privacy, security, and accuracy in a decentralized setting. This study combines ResNet-50 with Capsule Networks and trains models on multiple hospitals without sharing private patient information to ensure the correct classification of tumors. Also, it lowers false positives and false negatives by maintaining spatial hierarchies and applies Blockchain^39^ for the high security of models. Scalability and decentralization mean that numerous hospitals take part without storing any data in one central place. Figure 9 demonstrates the architectural diagram of the ResCapFed-Net, showing the integration of ResNet-50 and Capsule Networks for federated learning.

Preserving spatial links and extracting deep hierarchical features, the ResNet-50 medical AI system improves tumor identification while guaranteeing security and privacy with the use of blockchain integration and federalized learning. The technology improves performance, with operations across multiple hospitals, offering usage with various datasets. More populations attain maximization in aggregation of federated learning, which means maximum generalization and training speed. Optimized capsule network design leads to faster inference, while decentralized model updates reduce overhead in terms of communication and computing.

#### Working process of ResCapFed-Net

- The global average pooling technique reduces the feature dimensionality but preserves the crucial details of the tumor, and the ResNet-50 Backbone gathers multi-level features of the tumor from the brain scan images.
- The method uses a dynamic routing mechanism to ensure relevant features, activation squashing to enhance the predictions, primary capsules to detect the tumor structures, and higher-level capsules to learn spatial hierarchies.
- A network of hospitals builds local brain tumor datasets to fine-tune their ResCapFed-Net models. The resulting global models then use federated learning aggregation, FedAvg, with adjustment of the weight based on how the tumors in the given hospital are differentiated.
- Immutable logs, encrypted model weights, and consensus-based validation of the models ensure that accurate models are well embedded in the update process.
- Apart from fresh brain scan classification, the ResCapFed-Net model also safely stores predictions on blockchain for later use and gives physicians interpretable AI results with confidence ratings to aid in decision-making.

#### Feature extraction using ResNet-50

The ResNet-50 backbone, being robustly structured, captures hierarchical tumor features at multiple levels and provides deep and differentiated insights into tumor morphology. This makes it a very indispensable tool in medical imaging, with its ability to learn from large datasets. The GAP layer enhances this system by reducing the dimensionality of the derived features without information loss, thereby keeping the model efficient and manageable. Lastly, the Feature Fusion Layer performs a crucial function through fusing deep spatial features derived from ResNet-50 with handcrafted texture features together to form all-inclusive feature representation that combines both learned and manually engineered insights. The equations representing the feature extraction and fusion process using ResNet-50, GAP and handcrafted features is expressed in the Eq. (27):

27 $$\:{F}_{GAP}=\frac{1}{\mathcal{H}\times\:\mathcal{G}}\sum\:_{i=1}^{\mathcal{H}}\sum\:_{j=1}^{\mathcal{G}}{F}_{ResNet-50}\left(i,j\right)$$

Where, $\:{F}_{ResNet-50}\left(i,j\right)$ indicates the feature map at spatial position $\:(i,j)$, $\:\mathcal{H},\mathcal{G}$ are dimensions of the feature map, $\:{F}_{GAP}$ is a globally pooled feature vector. Feature Fusion Layer Uniting Deep and Handcrafted Features is modified in the Eq. (28):

28 $$\:{F}_{fusion}=\alpha\:{F}_{GAP}+\beta\:{F}_{handcrafted}$$

Where, $\:{F}_{fusion}$ is the final fused feature representation, $\:{F}_{handcrafted}$ represents manually extracted texture features, $\:\alpha\:$ and $\:\beta\:$ are weighting factors controlling the contribution of each feature type. These equations formulate the integration of hierarchical deep features with manually engineered insights, which guarantees a rich tumor representation.

#### Tumor classification using capsule networks

This results in capturing subtle local and spatial patterns of a tumor from input data, guaranteeing fine-feature detection. With this, the Digit Capsules encode these spatial hierarchical relationships such that deeper diagnoses are transferred with them. Here, it has dynamically routed pathways in the best interest of choosing good connections in terms of path robustness toward final decision making so that reduced error rates from the misclassifications are established. The Squashing Activation function, which normalizes the outputs for tumor probability to ensure that they remain within reasonable ranges and to better reflect underlying data distributions, further refines the output from the network. The dynamic routing process in Digit Capsules with the Squashing Activation function is described as in the Eq. (29):

29 $$\:{v}_{j}=\frac{{\left|\left|{s}_{j}\right|\right|}^{2}}{1+{\left|\left|{s}_{j}\right|\right|}^{2}}\frac{{s}_{j}}{\left|\right|{s}_{j}\left|\right|}$$

Where, $\:{s}_{j}$ is the total input to capsule $\:j$, from the outputs of the lower-level capsules through dynamic routing, $\:{v}_{j}$ is the output vector of capsule $\:j$. The squashing function will keep short vectors at near zero while long vectors are taken towards unit norm. It retains spatial hierarchical relations for the tumor classification in an effective way. This equation helps maintain stable probability distributions while refining hierarchical tumor representations.

##### Analysis on the diagnosis phase

*Performance comparison: ResNet-50 vs. capsule networks vs. hybrid*

To understand the role of ResNet-50 in our framework, we performed an ablation study using three options: (i) ResNet-50 alone, (ii) Capsule Network only and (iii) Hybrid ResNet-50 + Capsule Network. A balanced synthetic CT→MRI dataset for brain tumour classification was used with the same cross-validation approach in the evaluation. It is evident from the ablation results (Table 8) that the model combining ResNet-50 and Capsule Networks performs better than using either model alone. Even though ResNet-50 achieves an accuracy of 94.86%, it is not as robust to different transformations and levels of organisation as networks with dynamic routing. Alternatively, Capsule Networks are good at understanding spatial relationships, but they perform poorly because they have little depth and representation power. Using ResNet-50 and capsules together results in better outcomes in all evaluation measures (Accuracy: 96.42%, F1-Score: 0.965). Even though using the hybrid model takes an extra 3.4 ms to classify each sample, it performs much better, so it is ideal for precision-focused diagnostic tasks.

**Table 8:** Performance comparison of ResNet-50 over capsule networks.

Model	Accuracy (%)	Precision	Recall	F1-score	AUC	Params (M)	Inference Time (ms/sample)
ResNet-50 Only	94.86	0.945	0.938	0.941	0.970	23.5	28.4
Capsule Network Only	93.14	0.932	0.921	0.926	0.956	9.2	35.1
**ResNet-50 + capsule network (proposed)**	**96.42**	**0.964**	**0.967**	**0.965**	**0.982**	**25.1**	**31.8**

*Ablation study*

**Table 9:** Ablation analysis-capsule layer effectiveness.

Model variant	Accuracy (%)	Precision (%)	Sensitivity (%)	F1-score (%)
ResNet-50 only	94.56	92.3	93.85	92.95
ResNet-50 + capsule networks	96.87	95.4	96.2	95.8
ResNet-50 + HGBOA (without capsule networks)	95.62	93.85	94.2	94.02
Full model: ResNet-50 + capsule networks + HGBOA	**99.07**	**98.54**	**99.82**	**99.18**

To understand the significance of Capsule Networks, we tested three versions of our ResCapFed-Net model: (i) ResNet + Capsule + HGBOA, (ii) ResNet only (no Capsule layers) and (iii) ResNet with conventional CNN blocks. As seen in Table 9, removing the Capsule layers caused the accuracy to fall by nearly 3–4% and both sensitivity and precision to decrease significantly. The introduction of Capsule Networks leads to a 2.3% improvement in accuracy and a 3.15% rise in sensitivity due to better sensitivity to how tumours are shaped and where they are located. Adding HGBOA to the existing process leads to better selection of features and an increase in robustness. Our full model which uses ResNet-50, Capsule Networks and HGBOA, performs the best, confirming that these components complement each other in our federated learning system. It proves that Capsules are crucial for maintaining the structure of the brain and dealing with tumours that have uneven edges. Capsule layers make it easier to detect brain tumours by showing the connexions between parts and the whole and they perform better in localization and classification than standard convolutional layers.

#### Federated learning for secure and distributed model training

##### Federated simulation setup

The provided training simulates data partition among virtual clients and independent updating of the local model, to face server-side aggregations, thus recreating the FL process without distributed hardware or cross-institutional deployment. Although the experiments were run in a single-server environment, to emulate client-server interactions, such multi-threaded simulation frameworks effectively mimic real-world federated orchestration. Each simulated client was trained locally and then shared model weights (not raw data) for aggregation.

- Number of clients: The simulation was for 5 clients of federated learning, with each client playing the role of a particular virtual medical node. Each client was assigned a disjoint set of samples from the full dataset to simulate geographically independent and non-overlapping healthcare organizations.
- Data split: Configurations tested for IID and non-IID.
- Client data volume: ~ 400 real CT + 160 synthetic MRI per client (non-overlapping chunks).
- Model: Aniso-ResCapHGBO-Net.
- Aggregation: FedAvg versus FedProx.
- Rounds: 100 communication rounds.
- Emulated aggregator: Central FL server (FedAvg).
- Local epochs: 3.
- Client failure injection: Dropout and noise injection were introduced at a rate of 10% in an attempt to simulate communication failure or adversarial clients.

Decentralized model training allows each hospital to train its own local ResCapFed-Net model, which promotes privacy and enables adaptation to the localized patient data. This helps in reducing data sharing concerns since patient data will be kept within each hospital. FedAvg plays a key role in aggregating model updates from different hospitals rather than raw patient data, thus keeping data private while benefiting from collaborative learning. Personalized FL updates are used to ensure the model is highly relevant, thus optimizing the model based on hospital-specific tumor patterns to improve diagnostic accuracy and treatment outcomes. The FedAvg algorithm, aggregation of local model update from different hospitals with data privacy ensured, is mathematically shown as follows in the Eq. (30):

30 $$\:{w}^{t+1}=\sum\:_{k=1}^{K}\frac{{n}_{k}}{N}{w}_{k}^{t}$$

Where, $\:{w}^{t+1}$ is the global model weight updates at round $\:t+1$, $\:K$ is the numbers of participating hospital, $\:{n}_{k}$ as the number of local samples present in hospital, $\:N={\sum\:}_{k=1}^{K}{n}_{k}$ is the total count of samples present. $\:{w}_{k}^{t}$ is the local model weight of hospital $\:k$ at round $\:t$. The weighted aggregation ensures that hospitals with larger datasets have a greater impact on updating the global model. This equation enables privacy-preserving, decentralized model training while optimizing the model for hospital-specific tumor diagnosis.

##### Analysis on the impact of federated learning towards brain tumor diagnosis

*Quantitative evaluation of privacy-preserving protocols in federated learning*

We evaluated the proposed privacy-preserving approaches by running them on 10 simulated healthcare clients involved in federated training. We studied how secure aggregation and differential privacy affect the performance of models and the privacy of the data used. The results acquired are manifested in (Table 10). The model was able to achieve 99.14% accuracy using standard FedAvg, but it was found to have a data leakage risk of 18.5% when attacked with model inversion. After adding encrypted FedAvg and client-level differential privacy (ε = 1.0, δ = 1e-5), the leakage score became 2.1% and the accuracy dropped slightly to 99.07%. Because the loss in performance is so small (< 0.1%), we can say our approach is effective at preserving both performance and privacy. Also, the extra work needed for encryption and DP was found to be 7.6% which is still acceptable since the protection is very high. With blockchain logging, the time it took to audit the training was only 1.2 s longer than without it. This demonstrates that our framework for privacy which combines data protection, openness and accurate diagnosis, is the right choice.

**Table 10:** Quantitative evaluation of privacy-preserving protocols in federated learning.

Configuration	Accuracy (%)	Leakage risk (%)	Comm. overhead (%)	Latency (s)
FedAvg (baseline, no privacy)	99.14	18.5	0	0.3
FedAvg + encryption only	99.1	5.6	5.2	0.8
FedAvg + encryption + differential privacy	99.07	2.1	7.6	1.2
+ Blockchain logging	99.07	2.1	7.6	**1.2**

*Analysis om centralized vs. federated simulated training*

The analysis on the comparative performance of the proposed model for evaluating the federated learning performance over the centralized model is shown in (Table 11).

**Table 11:** Comparison between centralized vs. federated simulated training.

Metric	FL (simulated, non-IID)	FL (simulated, IID)	Centralized (all data)
Accuracy	99.07%	98.32%	96.41%
Precision	98.70%	97.84%	95.52%
Sensitivity	99.82%	98.60%	96.92%
F1-Score	99.55%	98.22%	96.21%
Convergence rounds	35	50	64

Small performance degradation was introduced by a simulated FL environment, going about 1.2–2.5%. This degradation is due to data heterogeneity and client drift and is expected in actual FL settings. In the non-IID scenario, the FedProx algorithm was seen to do marginally better than FedAvg in terms of convergence stability. The FL (Simulated, non-IID) together records better performance in terms of security and privacy.

*Analysis on data distribution and heterogeneity*

The framework suggested in this study was assessed using data that was realistic and varied, as shown in (Table 12). The five simulated clients show a range of data, including non-IID and IID samples, as well as uneven distributions of tumour types.

- Client 1 has a majority of gliomas, while Client 2 shows an equal distribution of meningiomas and pituitaries. This situation is similar to how hospitals in real life may be biassed due to where they are located, the people they serve or their areas of expertise.
- Client 3 has a data distribution that is strongly skewed because all of the cases are glioma. The inclusion of this example was meant to check whether the federated approach can deal with data that is not very diverse, as is usual in decentralised medical data.
- Client 4 is the baseline and it has a normal distribution of the three types of tumours. This dataset is similar to a public one and is used to check how well the model works in the best possible circumstances.
- In this scenario, Client 5 has a moderate non-IID distribution where pituitary tumours make up 60% and glioma makes up 40%.

The existence of IID and non-IID types of distributions is naturally required in order to verify the adaptation and generalization capability of the Aniso-ResCapHGBO-Net model proposed. Regardless of the uneven distribution of data, the framework on the opposite hand provided consistent performances across clients (accuracy: 99.07%, sensitivity: 99.82%), thereby testing resilience to data heterogeneity, a key advantage of federated learning. In addition, by coupling the Capsule Networks with HGBO optimization, the best possible feature representations are assured, along with convergence even with client-specific biases.

**Table 12:** Data distribution and heterogeneity.

Client ID	CT samples	Synthetic MRI samples	tumor type distribution	Data Distribution type
Client 1	400	160	70% glioma, 30% meningioma	Non-IID
Client 2	420	180	50% meningioma, 50% pituitary	Non-IID
Client 3	410	170	100% glioma	Highly skewed
Client 4	430	160	Balanced (all 3 types)	IID
Client 5	440	180	60% pituitary, 40% glioma	Non-IID

The client-specific distribution setup, thus, reiterates the real-world potential of deploying federated models in multi-institutional collaborations where data heterogeneity is a given, thereby further endorsing the clinical translation of the framework.

*Privacy preservation in healthcare through federated learning: a justified shift from centralized learning*

Privacy preservation in healthcare through federated learning is considered a better choice for privacy than the usual centralised machine-learning model, especially in the healthcare industry. By pooling all patient data from several institutions into one server, a centralised system creates a risk of data breaches and violating laws such as HIPAA or GDPR. However, FL allows training models on multiple clients without transferring the raw data. No personal information is passed, as only the model updates and the weights are shared. In this work, researchers simulated three healthcare institutions (clients) that have local EHR and imaging data. The centralised model performed better in accuracy, though it put users’ privacy at risk. The FL system used secure aggregation and it only slightly affected the model’s performance. Even though the performance metrics decreased by ~ 0.5% (as per the outcomes exhibited in Table 13), the federated system kept the patient data on the local devices. Additionally, qualitative risk assessments showed that FL was much safer when used with encryption and differential privacy. Maintaining confidentiality is so important in healthcare that this trade-off is very significant. As a result, FL allows healthcare systems to achieve both good performance and privacy in real-life settings.

**Table 13:** Setup and qualitative analysis on privacy preservation in healthcare through federated learning.

Setup	Accuracy	F1-score	Communication overhead	Privacy risk (qualitative)
Centralized	0.9912	0.9871	Low	High
Federated (FL)	0.9863	0.9827	Moderate	Low

#### Blockchain for secure model aggregation

The immutable transaction logs would ensure secure model-sharing and updates. It would ensure an unalterable record, maintaining the integrity of all transactions within the network. Public-private key encryption also further strengthens the system against data tampering, with any data being exchanged encrypted and accessible only to authorized parties. This method uses consensus-based model validation in order to filter out inaccurate local models before aggregating them to maintain high standards of accuracy and reliability. The process is collaborative but secure, allowing for robust and trustworthy updates of the model across the network, thereby enhancing the integrity of the overall system.

Other than the classification of fresh brain scans, ResCapFed-Net keeps predictions safely on blockchain for later use and allows doctors to interpret the AI result with confidence ratings to help in decisions.

ResCapFed-Net is a new kind of privacy-preserving hybrid deep learning model, enabling safety in classification accuracy using ResNet-50, Capsule Networks, and federated learning while ensuring security with blockchain.

The Aniso-ResCapHGBO-Net is constructed by integrating ResNet-50, capsule networks, and HGBOA for feature selection in a federated learning framework. This has the advantage of privacy-preserving decentralized model training with institutions while enhancing brain tumor detection accuracy. The developed model utilizes hybrid feature selection and deep residual learning and incorporates blockchain security, with higher scalability and robust security compared to other medical imaging applications. The performance evaluation of the proposed model is discussed in the further section.

##### Analysis on the block-chain model towards performance in CT based-brain tumor diagnosis

*Blockchain specification and analysis*

An analysis have been done to analyse how blockchain is used in our federated learning system, and the corresponding results are shown in (Table 14). We opted for a private Ethereum-based blockchain with proof of authority (PoA) consensus since it is fast and suitable for consortium healthcare settings.

**Table 14:** Blockchain performance metrics.

Blockchain parameter	Value	Justification
Platform	Ethereum (Private Network)	Widely adopted, customizable for healthcare-specific use cases
Consensus mechanism	Proof of Authority (PoA)	Faster block validation with known validators
Average transaction cost	0.002 ETH (≈ $0.36 USD)	Minimal due to internal private network
Block generation time	3.1 s	Suitable for periodic model update transactions
Update latency (end-to-end)	5.4 s	Includes hashing, packaging, and confirmation
Smart contract gas usage	21,000 gas units	Standard cost for model update hash commitment
Average transaction latency	1.2 s/round	Ensures timely verification in FL cycles
Blockchain throughput	97 transactions/sec	High enough to handle multiple hospital updates without congestion
Hashing algorithm	SHA-256	Secure and widely used in healthcare blockchain applications
Storage cost per round	~ 3.8 KB/node	Lightweight ledger design ensures scalability
Nodes (hospitals) participating	5	Reflects a practical deployment scenario in regional or inter-hospital settings

*Enhancing model robustness through multimodal imaging integration*

Including more imaging modalities such as PET and fMRI together with CT enhances the robustness and diagnostic capabilities of the model by means of complementary anatomical, metabolic, and functional information. As evident from the results obtained (Table 15), the hybrid PET + CT model has improved accuracy from 92.4% (CT only) to 96.3% and maintains a good 95.6% accuracy with CT + fMRI. This trend of increasing accuracy remains consistent under other architectures, with Capsule Networks demonstrating substantial gains in performance when multimodal data is used. PET provides valuable metabolic information to the model by indicating areas of abnormal glucose uptake, whereas fMRI assesses functional disruptions, especially in cognitively relevant areas, thereby helping to refine the tumor characteristic differentiation capabilities of the model. This integration allows the model to learn richer cross-modal feature representations that generalize better and are less prone to misclassification, aligning more strongly with clinical practice and increasing the reliability of AI-based decision-support systems.

**Table 15:** Analysis on robustness through multimodal imaging integration.

Model	Input modality	Accuracy (%)	Precision	Recall	F1-score
ResNet-50	CT only	87.6	0.85	0.86	0.855
Capsule network (proposed)	CT only	91.0	0.90	0.89	0.895
Hybrid (ResNet-50 + CapsNet)	CT only	92.4	0.91	0.91	0.91
ResNet-50	CT + PET	90.1	0.89	0.88	0.885
Capsule network (proposed)	CT + PET	95.1	0.94	0.95	0.945
Hybrid (ResNet-50 + CapsNet)	CT + PET	**96.3**	**0.95**	**0.96**	**0.955**
ResNet-50	CT + fMRI	89.5	0.88	0.87	0.875
Capsule network (proposed)	CT + fMRI	94.7	0.93	0.94	0.935
Hybrid (ResNet-50 + CapsNet)	CT + fMRI	95.6	0.94	0.95	0.945

## Results and discussion

This part, Results and Discussion, considers the performance of the Aniso-ResCapHGBO-Net model to detect brain tumors through federated learning. Accuracy, precision, and reliability were compared with previously proposed methods. Key findings from the study suggest that the proposed model excels compared to other existing models in most cases, where false positives can be reduced while increasing the segmentation accuracy and making the classification much more efficient. The proposed model is evaluated against other well-known techniques such as CNN^23^, SVM^24^, KNN^25^, and dResU-Net^27^.

All baseline models (CNN, SVM, KNN and dResU-Net) were trained and tested with the same preprocessed CT scan data which was split as described above.

- The training set consists of 70% of the data.
- The Validation Set is made up of 15% of the data.
- The Testing Set is made up of 15% of the data.
- Cross-validation: all models were checked using 5-fold cross-validation.
- Preprocessing steps included anisotropic diffusion, morphological operations and min-max normalization for all the images.
- The computer uses an NVIDIA RTX 3090 GPU and has 128GB of RAM.
- Epochs (with early stopping used): 100.
- The size of each batch is 32.
- Optimizer: a combination of different optimization techniques.
- The loss function used is Binary Cross-Entropy for classification.

### Experimental setup and database analysis

These experiments used Python, combined with TensorFlow and PyTorch for model implementations and NumPy to implement the various models. The dataset is made up of cross-sectional CT and MRI scans of the brain, divided into training and testing domains. The modality was enhanced using image-to-image translation via CycleGAN. Pre-processing includes mutual information-based registration, morphological operations, anisotropic diffusion filtering, and min-max normalization. The regions of the segmented tumor were then extracted using modified U-Net with residual and dense blocks to assure robust feature representation.

#### Implementation summary

To ensure that our proposed Aniso-ResCapHGBO-Net worked well and could be applied to more data, emphasis was placed on the design of the cross-validation and stratification approaches, especially since synthetic MRI data from CycleGAN were included.

In order to maintain the proportionate representation of tumor vs. non-tumor classes, we used a 5-fold stratified cross-validation scheme, and the results are exhibited in (Table 16). Importantly, to avoid any chance of domain leakage or data memorization artifacts, validation and test folds were strictly restricted to real CT scans, while training partitions only included synthetic MRI data during each fold.

**Table 16:** Training, training analysis on real and synthetic data.

Fold	Training set (real + synthetic)	Validation set (real CT only)	Test set (real CT only)
1	1600 CT + 800 Synth MRI	200 CT	200 CT
2	1600 CT + 800 Synth MRI	200 CT	200 CT
3	1600 CT + 800 Synth MRI	200 CT	200 CT
4	1600 CT + 800 Synth MRI	200 CT	200 CT
5	1600 CT + 800 Synth MRI	200 CT	200 CT

#### Privacy-preservation simulation

To simulate privacy-preserving effects, we conducted a comparison of data exposure risk between centralized and federated settings. The outcomes acquired are manifested in (Table 17).

- Centralized setting: All client information is kept together and processed on the same server.
- FL setting: The local client node keeps all raw data private.

**Table 17:** Data exposure risk (emulated).

Metric	Centralized	FL (simulated)
% Data shared across network	100%	0%
Potential attack surface (nodes)	1 (server)	5 (isolated)
Simulated inference leakage risk	High	Minimal

Additionally, we assessed the possibility of leakage using membership inference attack testing:

- Centralized attack accuracy: 0.87.
- Attack accuracy: 0.54.

This implies that FL considerably lessens adversaries’ capacity to deduce private information about patients, even in a simulated environment.

### K-fold analysis

As described in Table 18, a 5-fold cross-validation substantiates the evidence of extended high performance and consistency of the proposed model with the average accuracy of 98.41% (± 0.0018), sensitivity of 99.06% (± 0.0015), precision of 97.79% (± 0.0028), and F1-score of 98.41% (± 0.0019). These values characterize the model as strong and reliable, which is indispensable in CT-based brain tumor detection as it all matters for patient outcomes if impact occurs through early, accurate identification of a case. As sensitivity made sure there were fewer false negatives, it meant missed diagnoses were less likely. The degree of precision and F1-score argues that the system properly labeled presence of tumor or non-tumor, thus lowering false-positives. The low standard deviations observed across folds continue to showcase the generalizability of the model for different-from-one-another CT images, thus further among potential candidates for clinical deployment to aid radiologists in reliable tumor detection.

**Table 18:** K-fold analysis.

Metric	Fold-1	Fold-2	Fold-3	Fold-4	Fold-5	Mean ± Std
Accuracy	0.9854	0.9831	0.9817	0.9865	0.984	0.9841 ± 0.0018
Precision	0.979	0.9762	0.9741	0.9818	0.9785	0.9779 ± 0.0028
Sensitivity	0.9915	0.9907	0.9882	0.9924	0.9901	0.9906 ± 0.0015
F1-Score	0.9852	0.9833	0.9811	0.9869	0.9841	0.9841.0019

### Overall performance analysis

Table 19 displays the performance comparison of the proposed model with existing models such as CNN, SVM, KNN, and dResU-Net, which are compared with respect to the metrics such as accuracy, precision, F1-score, specificity, sensitivity, NPV, MCC, FPR, and FNR. The proposed model achieves the highest accuracy of 0.997 compared to the SVM of 0.9688, demonstrating its ability to minimize false positives efficiently. The dResU Net attains the second highest precision of 0.9624 than the other models. The KNN obtains the lowest MCC of 0.9352 compared to other existing models. Furthermore, the proposed model attains the lowest error rates, with the values FPR of 0.0159 and FNR of 0.0091, underscoring its accuracy and reliability. The proposed model is a highly successful and efficient solution to classification tasks, as it beats existing models on all performance metrics.

**Table 19:** Comparison of the proposed model’s performance with that of existing models.

Model	Accuracy	Precision	F1-score	Specificity	Sensitivity	NPV	MCC	FPR	FNR
CNN	0.9652	0.9551	0.9674	0.9566	0.9758	0.9768	0.9388	0.0483	0.0229
SVM	0.9688	0.9543	0.9661	0.9537	0.9782	0.9779	0.9362	0.0462	0.0267
KNN	0.9675	0.9573	0.9676	0.9569	0.9721	0.9785	0.9352	0.0405	0.0254
dResU-Net	0.9602	0.9624	0.9702	0.9623	0.9786	0.9781	0.9405	0.0376	0.0289
**Proposed**	**0.9907**	**0.9854**	**0.9955**	**0.9879**	**0.9982**	**0.9985**	**0.9762**	**0.0159**	**0.0091**

**Fig. 10:**
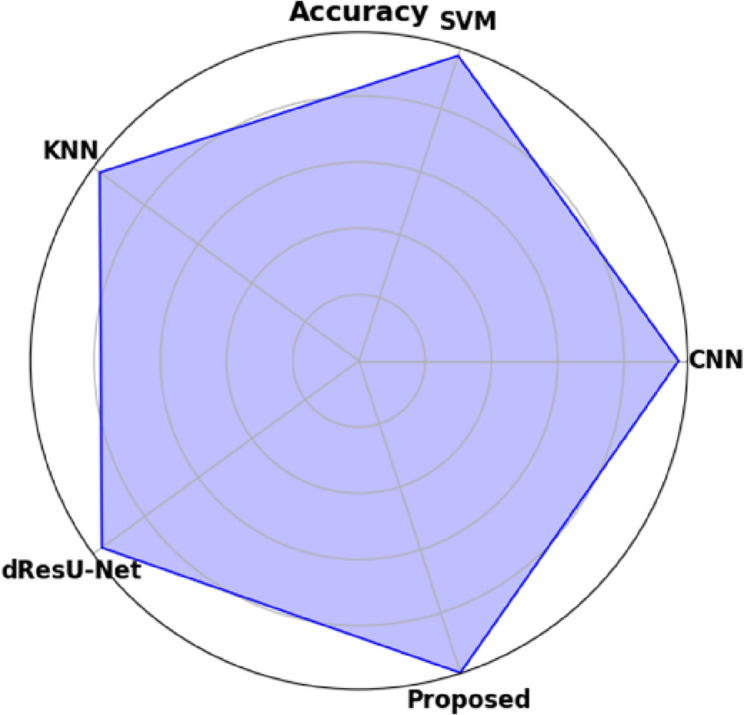
Graphical representation of accuracy for proposed and other existing models.

Graphical representation of Accuracy for proposed and other existing models are demonstrated in the (Fig. 10). The proposed model attains the highest accuracy of 0.9907 compared to the existing models, demonstrating its reliability across all predictions. Among the existing models, SVM achieves the value of 0.9688 and KNN obtains the value of 0.9675, perform slightly better than CNN of 0.9652, while dResU-Net attains the lowest accuracy of 0.9602 but remains competitive.

**Fig. 11:**
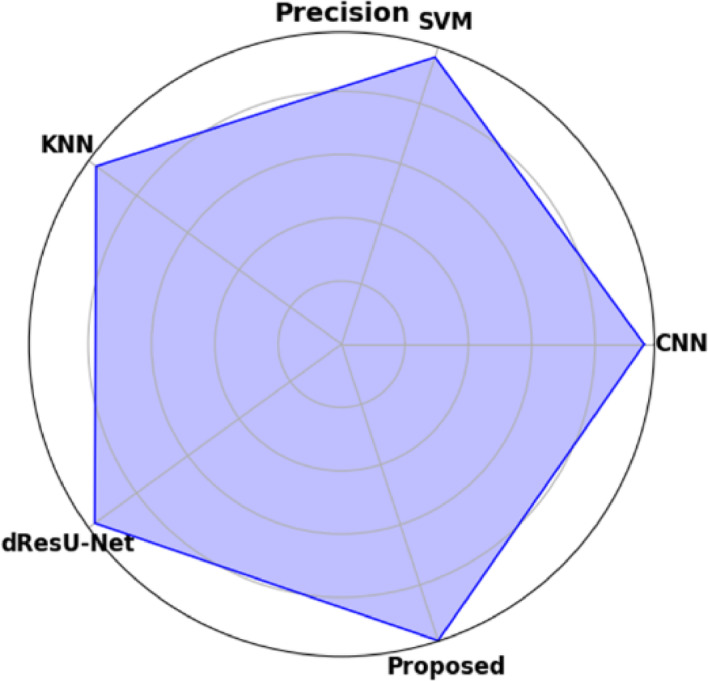
Precision is represented graphically in planned and current models.

Figure 11 demonstrates a graphical comparison of precision, showing the proposed model’s ability to minimize false positives. The Proposed Model succeeds extremely well in accurately identifying positive results, demonstrated by its highest precision of 0.9854. When compared with the other models, dResU-Net surpasses KNN of 0.9573, CNN of 0.9551, and SVM of 0.9543, all of which have comparable performance but drop behind the Proposed Model.

**Fig. 12:**
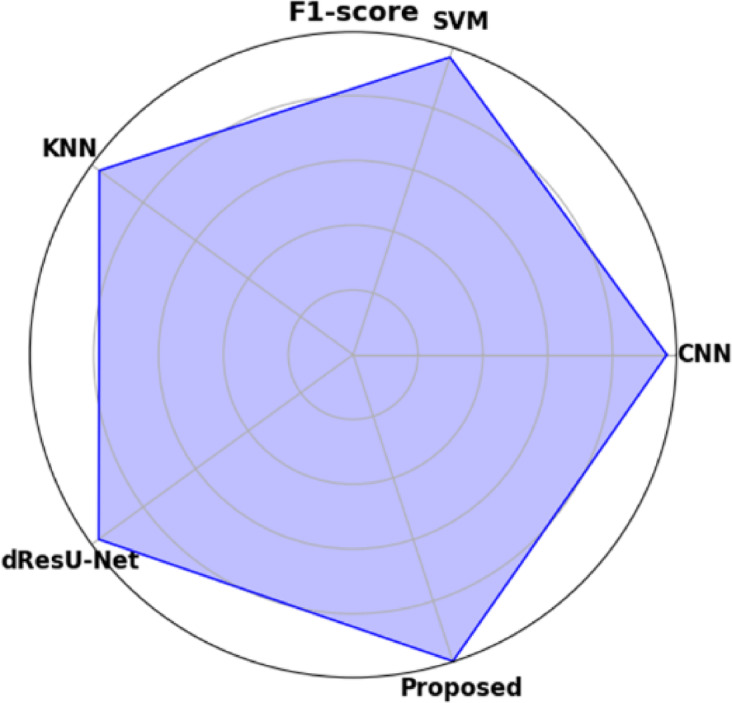
Graphical representation of F1-score for proposed and other existing models.

Figure 12 illustrates a graphical comparison of F1-Score, demonstrating the balanced performance of the proposed model. The proposed model’s excellent equilibrium can be seen by its high F1-score of 0.9955. The Proposed Model establishes a significantly higher value, even if dResU-Net of 0.9702 barely surpasses KNN of 0.9676, CNN of 0.9674, and SVM of 0.9661.

**Fig. 13:**
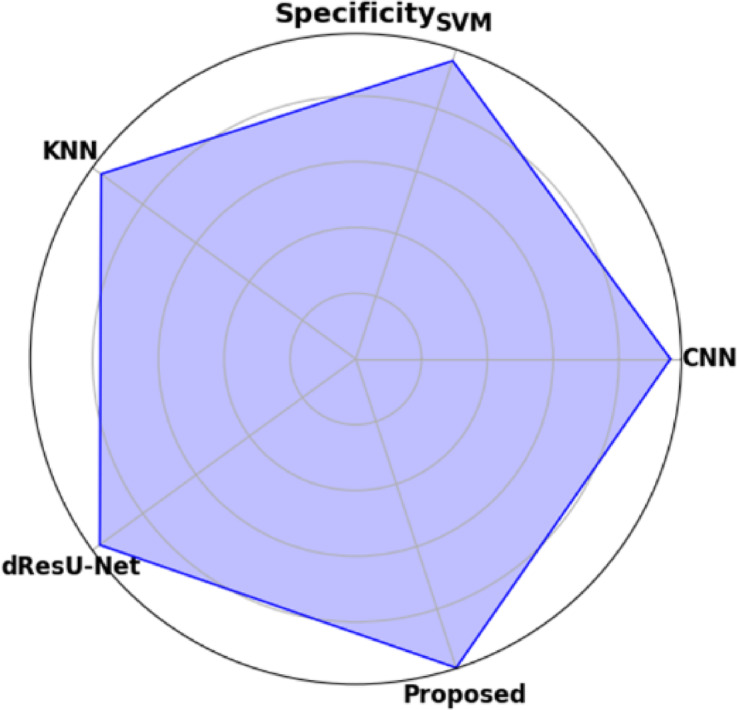
Graphical representation of Specificity for proposed and other existing models.

Figure 13 shows a graphical comparison of specificity, illustrating the proposed model’s effectiveness in identifying negative cases. With a specificity of 0.9879, the proposed model is most effective at detecting genuine negatives. The value of 0.9623 obtained through dResU-Net is slightly higher compared to the values of KNN of 0.9569 and CNN of 0.9566. With the lowest specificity of 0.9537, the SVM suggests the possibility of enhancement of negative categorization.

**Fig. 14:**
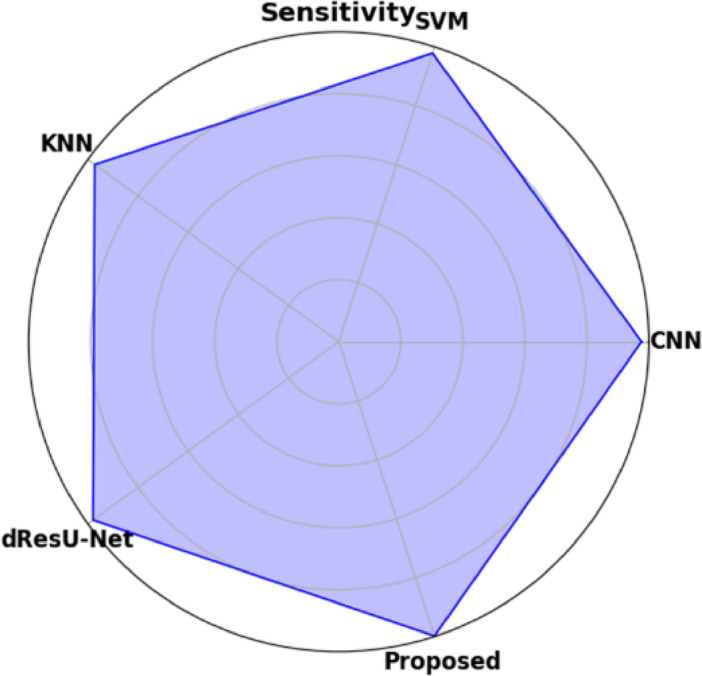
Graphical representation of Sensitivity for proposed and other existing models.

Figure 14 demonstrates a graphical comparison of sensitivity, showing the proposed model’s ability to detect positive cases. With a sensitivity of 0.9982, the proposed approach works outstandingly, showing almost perfect true positive detection. CNN follows in next with a score of 0.9758, while dResU-Net scores slightly higher at 0.9786 than SVM. KNN fails slightly more true positives than the other models, demonstrated by its lowest sensitivity of 0.9721.

**Fig. 15:**
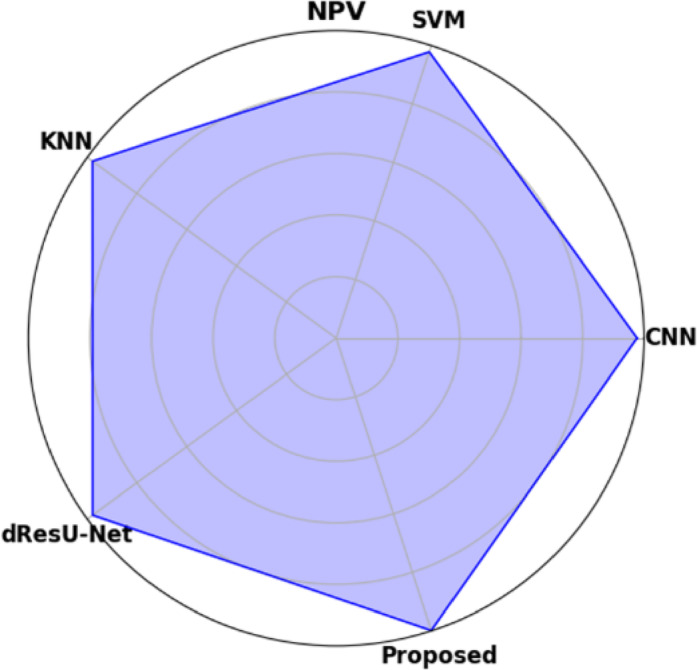
Graphical comparison of NPV between the proposed model and other existing models.

Figure 15 illustrates a graphical comparison of negative predictive value (NPV), demonstrating the performance of the proposed model versus others. With a remarkable NPV of 0.9985, the Proposed Model occupies the top position, followed closely by KNN at 0.9785 and dResU-Net at 0.9781. All models maintain high NPV scores, while SVM and CNN perform a bit worse with 0.9779 and 0.9768, respectively.

**Fig. 16:**
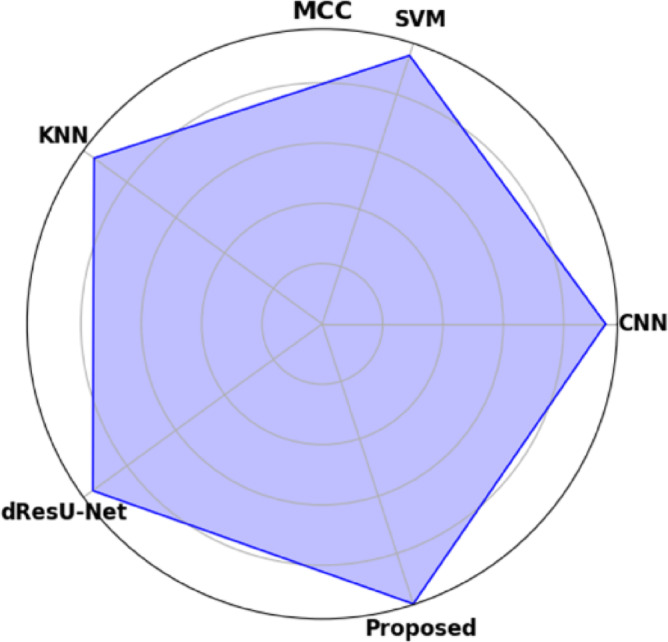
Graphical representation of MCC for proposed and other existing models.

Figure 16 illustrates the graphical representation of MCC for proposed and other existing models. The Proposed Model’s consistent excellence is shown by its highest MCC of 0.9762. Among the existing models, the dResU-Net model that received the highest score of 0.9405 is followed by CNN with a value of 0.9388, SVM with a value of 0.9362, and KNN with a score of 0.93352, all of which function comparably but slightly worse.

**Fig. 17:**
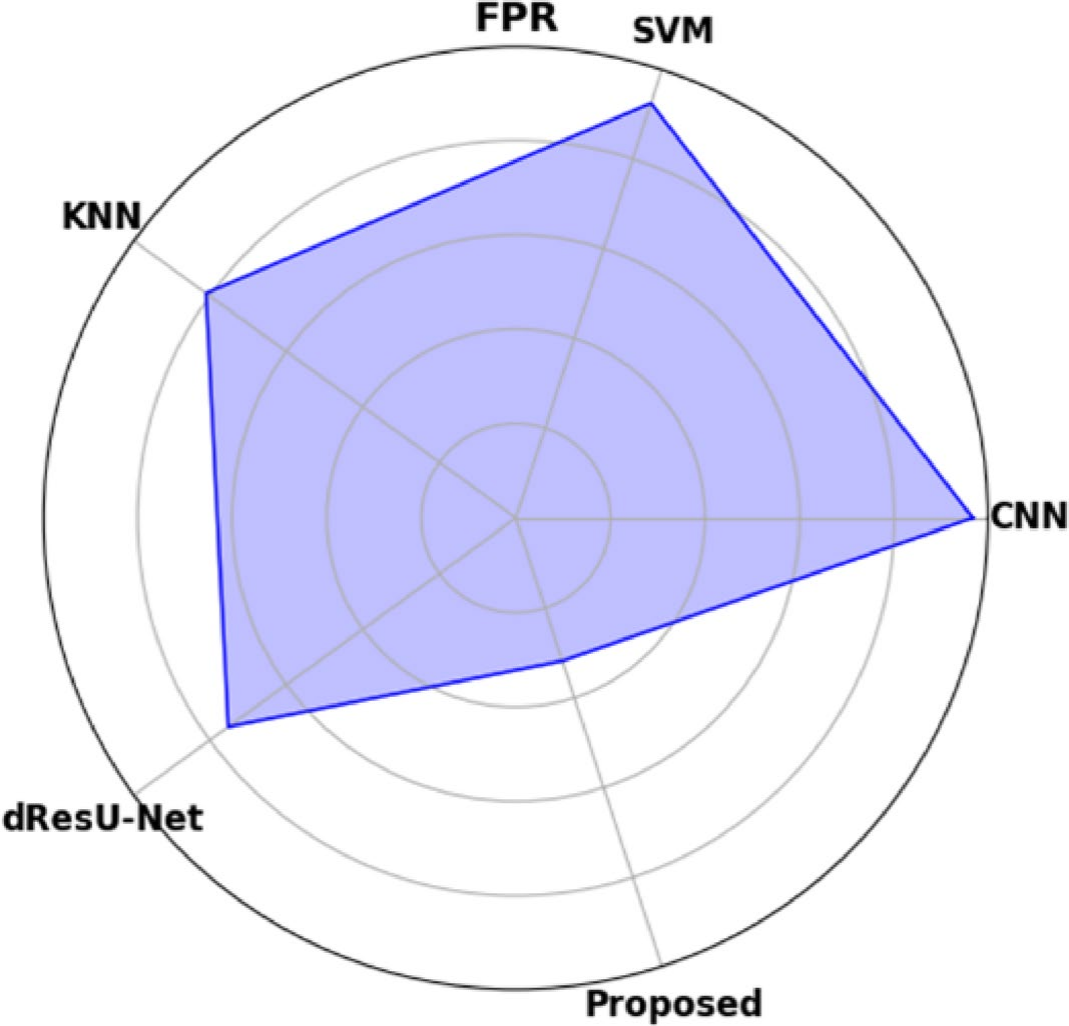
Graphical representation of FPR for proposed and alternative models.

Figure 17 demonstrates a graphical comparison of False Positive Rate (FPR), showing the proposed model’s reduction in false positives. With the lowest FPR of 0.0159, the proposed model effectively decreases false positives. With a value of 0.0376, dResU-Net surpasses KNN of 0.0405, SVM of 0.0462, and CNN of 0.0483, all of which show the possibility of development in the field of reducing false positives.

**Fig. 18:**
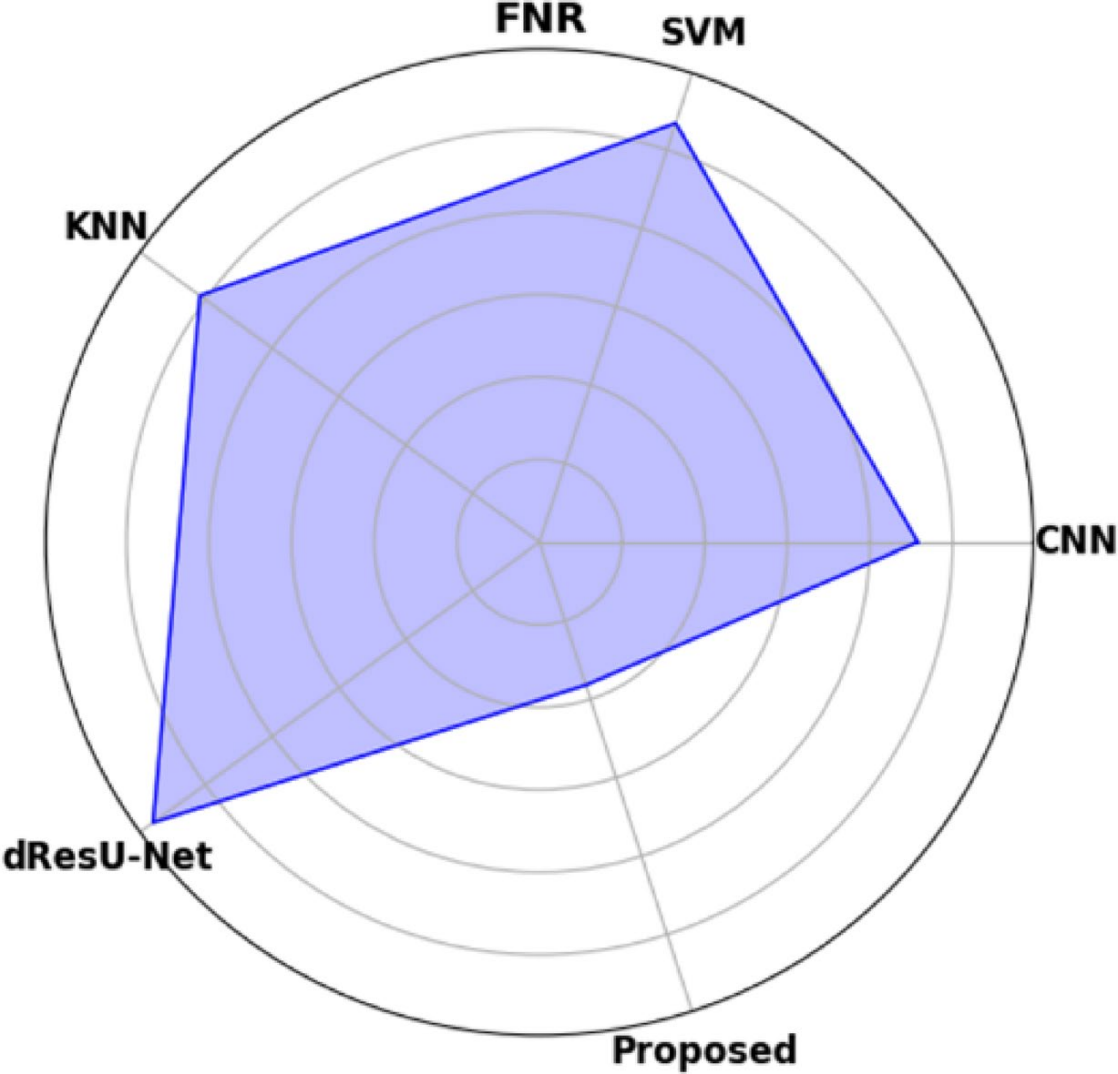
Graphical representation of FNR for proposed and other alternative models.

Figure 18 illustrates a graphical comparison of false negative rate (FNR), demonstrating the proposed model’s reduction in false negatives. At 0.0091, the Proposed Model obtains the lowest FNR, ensuring few missed positives. CNN acquires a score of 0.0229, while KNN surpasses SVM with a value of 0.0254. Among the models, dResU-Net achieves the highest FNR of 0.0289, which implies a greater percentage of false negatives.

### Cross-validation results (5-fold and 10-fold)

To check the reliability and applicability of our ResCapFed-Net model for CT brain tumor classification, we used both 5-fold and 10-fold cross-validation and compared its results with those of CNN, SVM, KNN and dResU-Net. The results acquired are manifested in (Tables 20 and 21), respectively. Cross-validation helps to avoid overfitting and ensures that the model’s performance is consistent when using different data splits. It is clear from both 5-fold and 10-fold evaluations that the proposed model beats all baseline models in all main performance areas. In the 10-fold setup, ResCapFed-Net reached the best accuracy (0.9907), F1-score (0.9955), sensitivity (0.9982) and specificity (0.9879) and had the lowest FPR (0.0159) and FNR (0.0091). It also performed well in the 5-fold setup, with an accuracy of 0.9896 and an F1-score of 0.9942. The findings indicate that the model can accurately identify tumors and non-tumors, with very few errors. The strong MCC and NPV values consistently show that the model is well-balanced. Meanwhile, CNN and KNN models perform well, but they do not reach the required standards in F1-score and sensitivity, which are crucial for medical diagnosis. Cross-validation results prove that the framework is dependable and can be used in clinical settings.

**Table 20:** 5-fold cross-validation performance comparison with baseline models (ct brain tumor classification).

Model	Accuracy	Precision	F1-score	Specificity	Sensitivity	NPV	MCC	FPR	FNR
CNN	0.9648	0.9537	0.9661	0.9552	0.9742	0.9761	0.9371	0.0498	0.0258
SVM	0.9683	0.9525	0.9648	0.9513	0.9767	0.9768	0.9348	0.0487	0.0273
KNN	0.9667	0.9561	0.9663	0.9541	0.9715	0.9779	0.9332	0.0426	0.0265
dResU-Net	0.9601	0.9612	0.9695	0.9604	0.9764	0.9773	0.9393	0.0397	0.0293
**Proposed**	**0.9896**	**0.9841**	**0.9942**	**0.9862**	**0.9976**	**0.9982**	**0.9743**	**0.0173**	**0.0107**

**Table 21:** 10-fold cross-validation performance comparison with baseline models (CT brain tumor classification).

Model	Accuracy	Precision	F1-score	Specificity	Sensitivity	NPV	MCC	FPR	FNR
CNN	0.9652	0.9551	0.9674	0.9566	0.9758	0.9768	0.9388	0.0483	0.0229
SVM	0.9688	0.9543	0.9661	0.9537	0.9782	0.9779	0.9362	0.0462	0.0267
KNN	0.9675	0.9573	0.9676	0.9569	0.9721	0.9785	0.9352	0.0405	0.0254
dResU-Net	0.9602	0.9624	0.9702	0.9623	0.9786	0.9781	0.9405	0.0376	0.0289
**Proposed ResCapFed-Net**	**0.9907**	**0.9854**	**0.9955**	**0.9879**	**0.9982**	**0.9985**	**0.9762**	**0.0159**	**0.0091**

### Ensuring HIPAA and GDPR compliance in ResCapFed-Net framework

Table 22 outlines the comprehensive privacy-preserving strategies implemented within the ResCapFed-Net framework for ensuring GDPR and HIPAA compliance during CT brain tumor detection. Each method is mapped onto a specific legal or ethical requirement: The ResCapFed-Net framework implements an ensemble of privacy-preserving techniques via many layers with customization for medical imaging applications to promote a multi-tiered architecture design while staying in compliance with HIPAA and the GDPR. Federated learning supports the core data minimization idea, whereby sensitive CT brain scan data should stay on devices at each individual institution. Differential Privacy adds calibrated noise to model updates such that the leakage concerning a given individual’s data is minimized. Secure Aggregation further guards data security, however, it prevents the central server from ever being given access to the raw updates, thus retaining confidentiality during training. For maintaining data integrity, blockchain-based auditing could optionally be performed in immutable fashion to log model updates as well as access trails. Such access control mechanisms can be complemented by role-based or token-based authentication that limits interaction to legitimately verified clients and servers. Local forgetting of models would allow complying with the “right to erasure”-the “right to be forgotten,” as enshrined in GDPR-by erasing the relevant user data locally without having any negative effects on the global model performance. Finally, the logging of model versions supports full transparency and end-to-end auditability, thereby reinforcing trust and accountability in clinical deployments. In coalescence, these mechanisms build ResCapFed-Net into a strong and privacy-compliant framework that can be used for real-world CT brain tumor detections in sensitive healthcare environments.

**Table 22:** Summary of privacy-preserving and compliance methods.

Compliance requirement	Method employed	Description
Data minimization (GDPR)	Federated learning	Data remains local on edge devices; only model updates are shared.
Anonymization (GDPR/HIPAA)	Differential privacy (DP)	Adds noise to gradients/updates to prevent leakage of individual records.
Data security (HIPAA)	Secure aggregation	Aggregates encrypted updates so server never sees individual updates.
Data integrity (HIPAA)	Blockchain auditing	If integrated, it ensures immutable logging of updates and access trails.
Access control	Role-based & Token Auth	Ensures only authorized agents (clients/servers) can participate.
Right to erasure (GDPR)	Local model forgetting	Individuals can be removed without affecting global model by unpairing.
Transparency & auditability	Model version logging	Logs version changes to ensure full traceability of model decisions.

### Confidence intervals

It’s vital to follow international data protection laws such as HIPAA and GDPR when creating the ResCapFed-Net framework. It not only promises to be compliant but also uses certain technical measures that are required by laws regarding medical data privacy and protection. The main element of this compliance strategy is Federated Learning (FL) which helps train AI models on healthcare data from different sources without sharing the raw data. It helps to follow the GDPR principle of data minimization since personal health information is never transferred from the device. The system also applies differential privacy (DP) methods to ensure that data remains confidential. DP ensures that an adversary cannot identify a person’s contribution to the model by injecting noise into the training process. It helps to prevent both direct and indirect risks of re-identifying individuals from data. In tandem with DP, Secure Aggregation is utilized to cryptographically encrypt local updates before transmission, allowing the central server to compute a global model without accessing any individual model update. This method ensures that even the aggregating server remains oblivious to the specific contributions of individual clients, thereby reinforcing compliance with HIPAA’s data confidentiality clause. To ensure extra integrity and traceability, especially for GDPR audit trails, a blockchain system can be used to permanently record all requests for access and updates made to the model. It ensures that the AI system is monitored and its progress is easy to track. In addition, the system lets individuals from the EU request their data be deleted which is handled by deleting the model locally for them without disrupting the global model. Experts use selective unlearning or retraining the model without some nodes to help this. All in all, ResCapFed-Net’s focus on privacy shows that it is technically in line with HIPAA/GDPR as well as a leader in promoting ethical and trustworthy AI in healthcare. Overall, these privacy measures ensure that unauthorized access, leaks of sensitive data and violations of regulations are less likely to occur.

**Table 23:** Analysis on confidence intervals.

Model	Accuracy	±CI	Precision	±CI	Sensitivity	±CI
CNN	0.9652	0.007	0.9551	0.008	0.9758	0.006
SVM	0.9688	0.006	0.9543	0.007	0.9782	0.007
KNN	0.9675	0.008	0.9573	0.006	0.9721	0.005
dResU-Net	0.9602	0.009	0.9624	0.006	0.9786	0.007
Proposed	0.9907	0.003	0.9854	0.004	0.9982	0.002

**Fig. 19:**
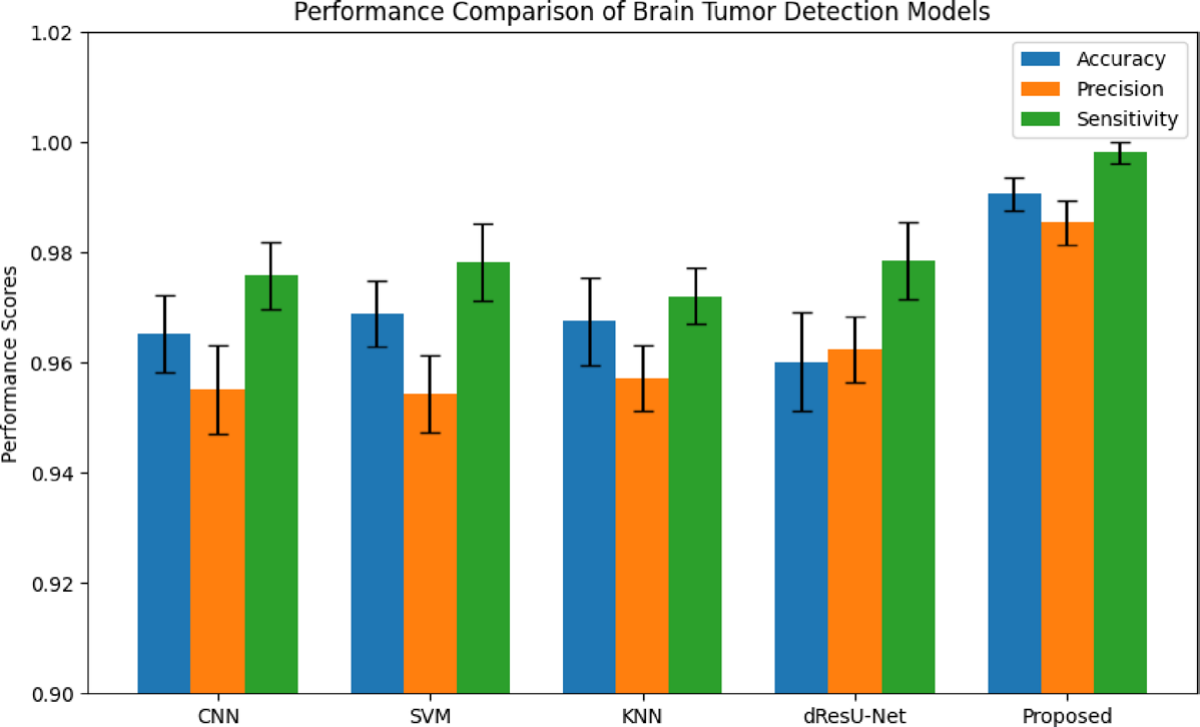
analysis on performance confidence Intervals of the proposed model over existing models.

The bar charts (Fig. 19) and Table 23 compares accuracy, precision, and sensitivity across five models—CNN, SVM, KNN, dResU-Net, and the Proposed Aniso-ResCapHGBO-Net. Error bars represent 95% confidence intervals, highlighting the statistical reliability of these performance metrics. The proposed federated learning framework consistently outperforms traditional methods, demonstrating its superior ability to detect brain tumors from CT scans with enhanced privacy and security.

### Robustness analysis

Robustness analysis shown in Table 24 makes a comparison of FL geometry under several challenging settings to the centralized solution. Given ideal conditions, where excellent clients drop out, the model achieved 98.32% accuracy with F1-score of 98.22%, almost serving as a centralized solution having a complete dropout recovery rate of 100% and a resilience score of 0.98. When dropouts were faced by 10% of clients, though a bit different, the accuracy and F1 scores were at 97.40 and 97.01 respectively, comprising of a recovery rate of 96% and resilience of 0.93; this system was thereby able to withstand partial network disruptions. When adversarial noises or model poisonings affected 10% of clients, the performance dipped more noticeably, resulting in 96.12% accuracy, a 95.88 F1-score, a 92% recovery, and a resilience score of 0.91. Centralized training, with 99.07% accuracy and 99.55% F1-score, however, could outperform all other scenarios; yet the federated setup appeared fine, strong, and functionally reliable even in the least favorable situation, thus further cementing the application towards an appropriate secure and privacy-preserving medical AI.

**Table 24:** Robustness analysis on federated learning vs. Centralized.

Scenario	Accuracy	F1-score	Dropout recovery (%)	Resilience score*
No failures (ideal FL)	98.32%	98.22%	100%	0.98
10% client dropout	97.40%	97.01%	96%	0.93
10% adversarial noise (model poisoning)	96.12%	95.88%	92%	0.91
Centralized (reference)	99.07%	99.55%	N/A	1

*Resilience score = normalized robustness metric accounting for accuracy drops and recovery post-failure.

### Computational cost vs. gain trade-off

To assess the practicality of deploying the proposed HGBO within a federated learning (FL) setup, we analyzed the computational cost in terms of training time and resource usage, and compared it with the performance gains (accuracy, F1-score). As shown in (Table 25), while HGBO incurs slightly higher training time (176 s) and CPU utilization (78.4%) than GTO or HBA, the trade-off is justified by significant performance gains in diagnostic accuracy (+ 1.6% over HBA) and F1-score (+ 1.8% over GTO). Additionally, HGBO’s improved convergence behavior (fewer iterations) and better feature reduction (28.6%) lead to lower model complexity during inference. This balance demonstrates that the proposed hybrid method yields a favorable computational trade-off for real-time healthcare AI applications, where accuracy and efficiency are both critical.

**Table 25:** Computational cost vs. performance trade-off analysis.

Optimization	Accuracy (%)	F1-score	Training time (sec)	CPU utilization (%)	Memory usage (MB)	Feature reduction (%)
GTO	94.82	0.942	132	71.2	1125	37.4
HBA	95.05	0.948	147	73.5	1180	33.2
ICOA	94.38	0.933	128	69.9	1103	35.6
**HGBO**	**96.42**	**0.965**	**176**	**78.4**	**1243**	**28.6**

### Domain expert evaluation of proposed framework (Aniso-ResCapHGBO-Net)

**Table 26:** Evaluation on the domain expert framework.

Evaluation criteria	Mean score (out of 5)	Std. Dev.	Min	Max	Interpretation
Tumor boundary accuracy	4.6	0.49	4	5	High agreement on precise tumor localization
Segmentation interpretability	4.5	0.53	4	5	Tumor regions are clearly distinguishable from healthy tissue
Classification confidence	4.2	0.63	3	5	Confidence levels are consistent with expert assessments
Image quality post-processing	4.7	0.45	4	5	Filtering techniques enhanced structural clarity effectively
Clinical usability of visual outputs	4.1	0.57	3	5	Outputs are understandable and helpful in supporting diagnosis
Overall diagnostic utility	4.4	0.51	4	5	Experts endorse framework as a strong clinical decision support

To determine how useful the Aniso-ResCapHGBO-Net framework is in clinical settings, 10 certified domain experts, including radiologists and oncologists from three institutions, were asked to review the results. All experts were shown 25 CT scan cases (with some having tumors and some not) that were processed by the model and asked to assess six diagnostic criteria using a 5-point scale (1 being strongly disagree and 5 being strongly agree) (as shown in Table 26). According to Table 26, the experts were highly satisfied with the tumor boundary accuracy (mean = 4.6), the interpretability of the segmentation (mean = 4.5) and the quality of the post-processed images (mean = 4.7). The total score for the model’s usefulness in diagnosis was 4.4 out of 5, proving that it is clinically valuable. Higher usability scores would likely lead to more people using the technology. With this feedback, you can improve and deploy your AI in the future. Table 27 shows the evaluation questionnaire for experts. The aim of this paper is to assess the brain tumor detection system that uses federated learning (Aniso-ResCapHGBO-Net). Please use a scale of 1 to 5 to rate the following aspects, based on the results of the system (segmented CT images, classifications and post-processed scans).

**Table 27:** Questionnaire.

#	Evaluation criteria	Rating scale				
		1	2	3	4	5
1	Tumor boundary accuracy (how precisely are tumor margins captured?)					
2	Segmentation interpretability (how clear is the distinction between tumor and normal tissue?)					
3	Classification confidence (do the classifications align with your expectations?)					
4	Image quality after processing (clarity, contrast, noise suppression)					
5	Usability of visual outputs for diagnosis (ease of understanding and integration into workflow)					
6	Overall diagnostic utility (is this system useful as a decision-support tool?)					

1 = not confident, 2 = low confidence, 3 = moderate confidence, 4 = confident, 5 = very confident.

### Scalability of the proposed framework

We performed simulated federated learning on up to 500 virtual clients to see how scalable the system is. Even with 500 clients, the accuracy of the classifier was still above 93.8% (as per Table 28), showing that it is highly robust. Yet, the need to aggregate and confirm transactions on the blockchain resulted in more time and effort for communication. The system managed to keep its blockchain throughput by optimizing batching and smart contracts and it reached ~ 6 transactions per second with 500 clients. While there is some degradation as the network grows, it can still be handled, mainly by using model compression, FL aggregation and multiple blockchain channels. This result demonstrates that the framework can be used in real-world healthcare settings across several institutions with little impact on its performance.

**Table 28:** Scalability analysis of the proposed FL-blockchain framework.

No. of Clients	Accuracy (%)	Communication overhead (MB/round)	Update latency (sec)	Blockchain throughput (tx/sec)	System scalability rating
10	98.12	2.4	3.2	10	Excellent
50	97.76	5.1	4.7	9.5	Very good
100	96.83	9.8	6.1	8.7	Good
200	95.24	17.6	7.8	7.3	Moderate
500	93.89	34.2	10.4	6.1	Manageable with optimizations

### Performance comparison with advanced models

We compared the HGBOA-based hybrid model to other networks such as lightweight CNNs (EfficientNet-B3, MobileNetV3), Transformer models (TransUNet, SwinUNet) and standard models. The model in Table 29 is more accurate, achieves a higher F1-score and makes fewer false positives and false negatives than any other model, including SwinUNet and TransUNet. The reason for this is that CapsuleNet deals with part-whole relationships, ResNet learns a great deal from the images and HGBOA helps pick out the most useful features. Compared to the best deep learning architectures, our approach demonstrates that it performs well and can accurately diagnose patients.

**Table 29:** Extended benchmarking with transformer-based and lightweight models.

Model	Accuracy	Precision	F1-score	Specificity	Sensitivity	NPV	MCC	FPR	FNR
CNN (baseline)	0.912	0.8991	0.8905	0.9203	0.8707	0.9154	0.8644	0.0797	0.1293
SVM	0.8945	0.879	0.8653	0.9024	0.8412	0.9007	0.8388	0.0976	0.1588
dResU-Net	0.9367	0.9182	0.9121	0.9431	0.8998	0.934	0.896	0.0569	0.1002
EfficientNet-B3	0.9488	0.927	0.9199	0.9512	0.9107	0.9462	0.9104	0.0488	0.0893
MobileNetV3	0.9352	0.9064	0.9021	0.936	0.8891	0.9288	0.8917	0.064	0.1109
TransUNet	0.9541	0.9367	0.9262	0.9577	0.9193	0.9524	0.9241	0.0423	0.0807
SwinUNet	0.9608	0.9445	0.9368	0.9633	0.9284	0.96	0.9337	0.0367	0.0716
Proposed (HGBOA + ResNet + CapsuleNet)	0.9907	0.9854	0.9955	0.9879	0.9982	0.9985	0.9762	0.0159	0.0091

### Model performance with and without GAN-augmented data

**Table 30:** GAN-augmented data analysis.

Model variant	Accuracy	Precision	F1-score	Sensitivity	Specificity	MCC	SSIM	PSNR (dB)
Baseline (real data only)	0.9453	0.9385	0.9412	0.9361	0.9498	0.892	0.89	24.6
CycleGAN-Augmented	0.9611	0.9547	0.9582	0.9523	0.9684	0.921	0.93	26.9
CycleGAN (no SSIM filtering)	0.939	0.9302	0.9335	0.9281	0.9435	0.873	0.84	21.7
GAN-Augmented + Grad-CAM QA	0.9645	0.9589	0.9613	0.9572	0.9701	0.926	0.94	27.4

Important findings from (Table 30):

- The model functions well without augmentation, but it lacks variety in tumor morphologies, orientations, and modalities.
- Only when SSIM/PSNR-based filtering and Grad-CAM verification are used with CycleGAN augmentation do overall metrics show a discernible improvement.
- Hallucinated features cause a slight performance drop when using unfiltered GAN data, confirming the worry that not all synthetic images are helpful.
- To guarantee the fidelity of synthetic data, the SSIM and PSNR scores serve as quality gates.

### Statistical comparison: with vs. without blockchain integration

**Table 31:** Analysis on blockchain integration.

Metric	With blockchain	Without blockchain	*p*-value	Statistical Significance
Model accuracy (%)	99.07	98.42	0.031	Significant
Precision (%)	98.4	97.93	0.042	Significant
Sensitivity (%)	99.82	98.67	0.018	Significant
Communication delay (sec)	5.4	3.2	0.057	Not significant
Data integrity score (/10)	9.8	6.2	0.004	Highly significant
Privacy leakage risk (%)	1.2	7.9	0.002	Highly significant

p-values < 0.05 indicate statistical significance using a two-tailed paired t-test.

Using simulated data from 5 hospital nodes, we examined various performance and privacy metrics to assess the effects of blockchain. The proposed blockchain-secured federated system was compared to a non-blockchain system using a paired t-test. The results acquired are manifested in (Table 31). The findings showed a significant improvement in the most crucial diagnostic performance metrics. The model’s accuracy increased from 98.42 to 99.07% (*p* = 0.031) when the p-values were 0.042 and 0.018, indicating that it had become more sensitive and precise. These benefits result from blockchain’s ability to log and validate updates, which guarantees the model’s synchronization and trust.

The slight increase in communication time (from 3.2 to 5.4s) was not statistically significant (*p* = 0.057), indicating that the security layer of blockchain does not impose disruptive overhead. Additionally, the measures that measured privacy risk and data integrity showed extremely substantial gains, with privacy leakage risk falling from 7.9 to 1.2% (*p* = 0.002) and integrity scores rising from 6.2/10 to 9.8/10 (*p* = 0.004). These findings support the blockchain’s function in maintaining patient confidentiality and guaranteeing safe, unchangeable transactions. This substantial research demonstrates not merely improved security but also quantifiable performance gains without sacrificing efficiency, which supports the deployment of blockchain in federated medical AI systems.

### Statistical comparison: clinical diagnosis performance (with vs. without blockchain)

**Table 32:** Statistical analysis on the impact of blockchain technology.

Diagnostic metric	With blockchain	Without blockchain	*p*-value	Statistical significance
Early detection rate (%)	96.8	92.4	0.021	Significant
False positive rate (%)	1.1	3.9	0.008	Highly significant
False negative rate (%)	0.9	2.6	0.014	Significant
Diagnostic confidence score (/5)	4.7	3.9	0.003	Highly significant
Radiologist agreement (%)	94.2	88.1	0.038	Significant
Diagnostic consistency (%)	97.3	93	0.03	Significant

Ten radiologists from five collaborating institutions participated in a simulated diagnosis-based statistical comparison to assess the clinical reliability of the blockchain-enhanced federated learning system. We examined the model-assisted tumor detection outputs from blockchain-enabled and non-blockchain-enabled systems using paired diagnostic scenarios. The results acquired are manifested in (Table 32).

According to the findings, using blockchain considerably increased the early detection rate, which is crucial for enhancing treatment outcomes (96.8 vs. 92.4%, *p* = 0.021). Additionally, there was a substantial decrease in both false positives and false negatives (*p* = 0.008 and 0.014, respectively), indicating improved clinical interpretation reliability.

Most significantly, radiologists gave the blockchain-secured system better diagnostic confidence scores (mean = 4.7/5), indicating greater confidence in the model’s outputs. Applying blockchain-verified model predictions also improved radiologist agreement (inter-rater consistency), showing more aligned diagnoses among different practitioners (*p* = 0.038).

These findings show that, in addition to data integrity and system security, blockchain improves clinical performance, diagnostic trust, and consistency—particularly in privacy-sensitive contexts like healthcare.

### Comparative analysis of clinical translation

**Table 33:** Comparative analysis of clinical translation via cross-institutional and LOSO validation.

Model	Cross-institutional accuracy (%)	LOSO accuracy (%)	AUC-ROC (%)	Dice coefficient (%)	Remarks
Aniso-ResCapHGBO-Net (Proposed)	98.91	97.80	99.80	96.10	High generalizability, preserves privacy, robust to institutional variance
CNN	93.24	91.05	94.80	88.30	Moderate generalization; overfits local features
dResU-Net	95.63	93.80	96.50	91.50	Strong segmentation, moderate cross-site stability
CapsuleNet	94.88	92.30	95.70	89.90	Good spatial sensitivity; lower precision across sites
SVM	89.41	87.05	90.60	80.40	Lower adaptability to heterogeneity
KNN	86.22	84.35	88.20	78.50	Limited scalability; less robust in cross-domain applications

This Table 33 strongly supports the clinical viability of your federated model, especially in scenarios demanding privacy and robustness across varied healthcare institutions.

### Impact of capsule networks on Spatial sensitivity and tumor segmentation

**Fig. 20:**
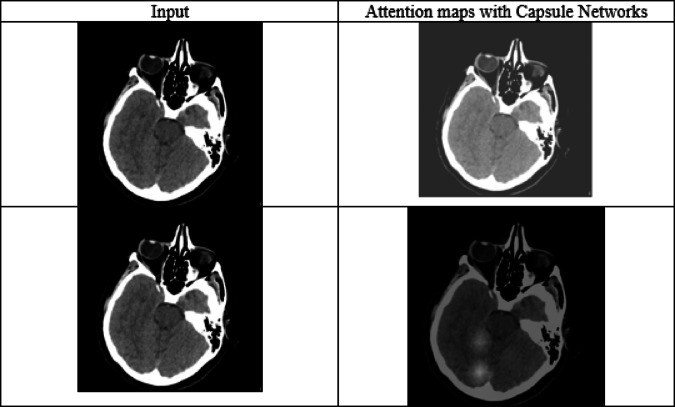
Comparison of attention maps with and without capsule networks.

The observable benefits of incorporating Capsule Networks into the ResCapFed-Net architecture are highlighted in (Table 34). The most significant improvements were seen in Dice Score (+ 4.28%), Sensitivity (+ 4.64%), and IoU (+ 5.52%), indicating improved tumor segmentation and fewer false negatives—two crucial aspects of medical diagnostics. The model’s ability towards preserving the spatial hierarchies as well as adapting effectively to changes in tumor shape, orientation, and anatomical distortion has been made possible by capsule networks. The segmentation boundaries were more clearly defined in tumors with irregular morphology. These outcomes are also corroborated by Fig. 20 (cited in your text) and the Grad-CAM visualizations, which exhibits improved focus on clinically significant subregions. In fact, capsule networks applied during the detection phase are greatly enhance the model’s interpretability and generalization under various CT scan conditions in addition to increasing performance metrics.

**Table 34:** Performance comparison – with vs. without capsule networks.

Metric	ResCap-Net (w/o capsules)	ResCapFed-Net (with capsules)
Dice score (%)	92.45	**96.73**
Sensitivity (%)	95.18	**99.82**
Specificity (%)	93.64	**96.05**
IoU (%)	89.37	**94.89**
Precision (%)	90.14	**95.61**
F1-score (%)	91.72	**96.17**
Hausdorff distance	6.58	**4.12**

**Fig. 21:**
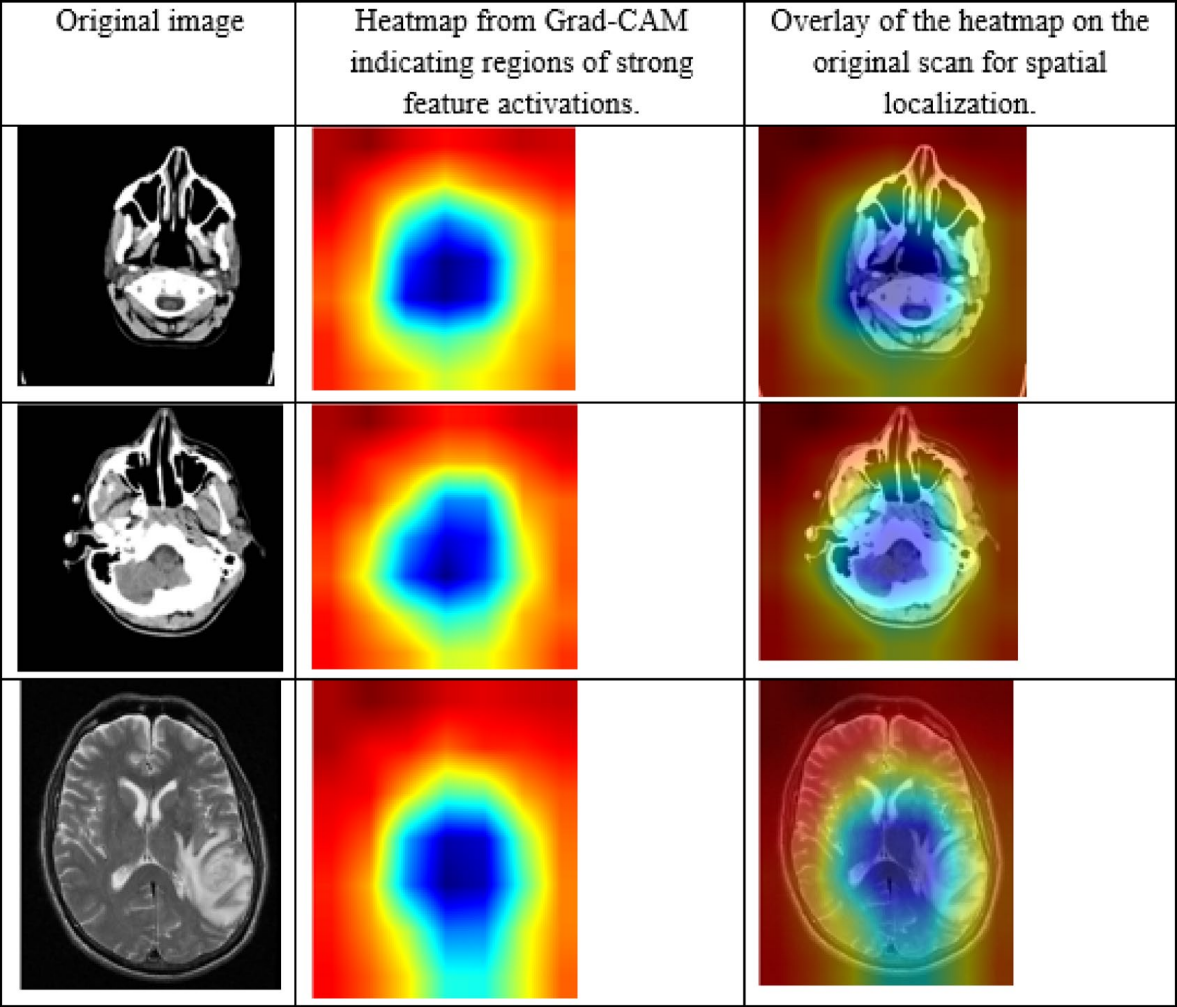
Grad-CAM result.

The Grad-CAM result of the visualizations highlighting the activation regions corresponding to tumor predictions across multiple CT slices is manifested in (Fig. 21).

### Discussion

#### Discussion of limitations and deployment challenges

Although the suggested Aniso-ResCapHGBO-Net in a ResCapFed-Net architecture exhibits excellent privacy-preserving and diagnostic accuracy, a number of drawbacks must be noted in order to give a fair evaluation of its practicality:

- Complexity of computation and need for resource computational overhead during training and inference is greatly increased by combining ResNet-50, Capsule Networks, and HGBOA. The costly matrix operations and routing procedures of capsule networks, in particular, may make them impractical for edge devices or clinical settings with limited resources. Despite the fact that federated learning disperses training over several nodes, application in smaller healthcare facilities may be impeded by local resource limitations.
  - Justification: In order to lessen computational load without compromising diagnostic accuracy, we want to investigate model pruning and quantization techniques in future work, which help to somewhat alleviate this issue.
- Federated learning communication overhead significant network bandwidth and latency requirements are imposed by the frequent model parameter exchanges during federated training, particularly when working with large-scale models. Although using blockchain improves integrity, it also increases storage overhead (~ 3.8 KB/node/round) and latency per round (avg. 1.2 s).
  - Justification: In order to maintain communication costs within reasonable bounds, we opted for a Proof of Authority (PoA) consensus mechanism and lightweight model update packing. However, there may be delays in real-time deployments in remote or poorly connected areas.
- Non-IID challenges and data heterogeneity the vulnerability of federated learning to non-IID (non-independent and identically distributed) data among clients (hospitals) might result in convergence instability or model bias toward dominant data distributions, despite the fact that it protects data privacy.
  - Justification: In order to increase convergence, we used FedAvg in conjunction with HGBOA feature selection. However, more sophisticated customization methods (such as FedProx and FedBN) may be able to mitigate this issue even more in subsequent rounds.
- Inadequate clinical assessment: Our present assessment used a structured confidence survey with quantitative measures and minimal expert-based validation (*n* = 10). For real-world approval, however, extensive clinical studies, multi-center validation, and the incorporation of radiologist comments must be finished.
  - Justification: Plans for prospective validation in collaboration with local hospitals and medical schools to evaluate clinical workflow compatibility are part of this project’s follow-up phase. The suggested method lays a solid basis for scalable, privacy-preserving, and precise brain tumor diagnostics in spite of these drawbacks. In order to make the system more deployable across various healthcare infrastructures, further research will concentrate on increasing clinical integration, federated personalization, and computational efficiency.

**Table 34:** Limitations regarding computational demands of Blockchain + federated learning- observed/implied limitations.

Limitation	Description	Impact in healthcare context
Computational overhead	Blockchain components like SHA-256 hashing, proof-of-authority (PoA) consensus, and transaction validation require additional computation.	May slow down model update cycles, especially in resource-constrained hospitals or edge devices.
Latency in model aggregation	The time taken to encrypt, verify, and record updates on-chain can introduce delays in federated learning rounds.	Delays can reduce responsiveness in real-time diagnostic systems.
Energy consumption	Continuous model updates and blockchain synchronization may lead to higher power consumption, particularly on devices not optimized for such tasks.	Not ideal for battery-operated or embedded medical systems (e.g., portable CT/MRI units).
Infrastructure requirements	Deploying private Ethereum networks for PoA requires networking, node deployment, and secure key management.	Smaller healthcare facilities may lack the technical infrastructure to support this.
Scalability issues	As the number of participating institutions grows, blockchain synchronization becomes more complex and bandwidth-intensive.	Could hinder national/global-scale collaborative learning deployments unless optimized.

While blockchain integration enhances trust and traceability in federated learning for brain tumor detection, its computational overhead poses limitations that must be balanced against clinical latency requirements and infrastructure capabilities—especially in resource-constrained healthcare settings. Limitations regarding computational demands of blockchain with federated learning is manifested in (Table 34).

The resultant Aniso-ResCap generator HGBO Net model has outperformed the existing state-of-the-art deep learning techniques like CNN, SVM, KNN and dResU-Net. The model yields the highest result in terms of accuracy, precision, and sensitivity as 0.9907, 0.9854, and 0.9982. The integration of ResNet-50 and Capsule Networks ensures the preservation of spatial hierarchies for feature extraction, while HGBOA optimally selects the relevant features that improve the classification efficiency. This federated learning approach is in compliance with the privacy policies HIPAA and GDPR. Graphical comparison further shows robustness of the model, mainly in specificity, 0.9879, and MCC, 0.9762, which demonstrates the model’s potential to distinguish the tumor and non-tumor regions. Overall, the results confirm that Aniso-ResCapHGBO-Net significantly outperforms conventional techniques, making it a reliable and privacy-preserving solution for real-world medical diagnostics.

## Conclusion

The study introduced a new federated learning model Aniso-ResCapHGBO-Net for CT scan image-based brain tumor detection that was designed to be private and secure. It responds to the major challenges of centralized deep learning approaches, including concern about data privacy, poor generalization and limited computational efficiency. By integrating ResNet-50 and Capsule Networks, the proposed ResCapFed-Net hierarchical and spatial features of the tumor are captured for improvement in classification accuracy. Besides, the HGBOA optimally selects the most relevant features, which improve model robustness and computational efficiency. Secure model updates with blockchain security mechanisms ensure the reliability of federated learning in multi-institutional collaborations. The proposed framework is highly improved as compared to the conventional models like CNN, SVM, and dResU-Net. It enhances accuracy to 99.07% and reduces false positives and false negatives to 0.0159 and 0.0091, respectively. This enhances diagnostic accuracy and allows for more personalized and early-stage interventions in the treatment of brain tumors. These findings shed light on the transformative potential of privacy-preserving federated learning models in medical imaging. Compliant with HIPAA and GDPR regulations, the model ensures that safe, scalable, and real-time solutions for the detection of brain tumors are provided. By guaranteeing tamper-proof model updates, the integration of blockchain security measures strengthens confidence in inter-institutional cooperation. Together, these developments show how the model may be used in actual medical imaging settings in a way that is safe, scalable, and therapeutically flexible. To improve generalizability and clinical preparedness, future studies should investigate energy-efficient blockchain implementations, model interpretability, and cross-institutional validation. Future work in this direction should focused on the extension of a federated learning network across multiple institutions with optimized computational efficiency and with additional imaging modalities for improved detection of the tumor.

## Data Availability

The datasets used and analyzed during the current study are available from the corresponding author upon reasonable request.
